# Clinical and genetic characterization of a large cohort of patients with Wilson’s disease in China

**DOI:** 10.1186/s40035-022-00287-0

**Published:** 2022-02-28

**Authors:** Shijie Zhang, Wenming Yang, Xiang Li, Pei Pei, Ting Dong, Yue Yang, Jing Zhang

**Affiliations:** 1 Experimental Center of Clinical Research, The First Affiliated Hospital of Anhui University of Chinese Medicine, Hefei, 230031 China; 2 Department of Neurology, The First Affiliated Hospital of Anhui University of Chinese Medicine, Hefei, 230031 China; 3 Key Laboratory of Xin’An Medicine, Anhui University of Chinese Medicine, Hefei, 230031 China

**Keywords:** Wilson’s disease, Chinese, *ATP7B*, Genotype–phenotype correlation, Large cohort study

## Abstract

**Background:**

Wilson’s disease (WD) is an autosomal recessive disorder of copper metabolism caused by *ATP7B* (encoding a copper-transporting P-type ATPase) variants that shows various characteristics according to race and geographical region. This study was aimed to provide a comprehensive analysis of *ATP7B* variants in China and to investigate a plausible role of common variants in WD manifestations.

**Methods:**

A total of 1366 patients (1302 index patients and 64 siblings) clinically diagnosed with WD (Leipzig score ≥ 4) were recruited. They underwent *ATP7B* gene sequencing and information of age and symptoms at onset was collected. The genotype–phenotype correlation was assessed in the index patients who were examined with two pathogenic variants and onset with hepatic (*n* = 276) or neurologic (*n* = 665) symptoms.

**Results:**

We identified 294 potentially pathogenic *ATP7B* variants (112 truncating, 174 missense, 8 in-frame) in the 1302 index patients, including 116 novel variants. The most frequent variant was c.2333G>T (R778L, allele frequency: 28.96%), followed by c.2975C>T (P992L, 13.82%), c.2621C>T (A874V, 5.99%), c.2755C>G (R919G, 2.46%), and c.3646G>A (V1216M, 1.92%). In 1167 patients, both pathogentic variants were identified, of which 532 different variant combinations were found. By binary logistic regression analysis, the factor associated with neurological presentation was high age-at-onset, but not sex, protein-truncating variant (PTV), or the common missense variants (R778L, P992L, and A874V). In the neurological group, low age-at-onset was a factor associated with dystonia, gait abnormality, and salivation; high age-at-onset was a factor associated with tremor; and the sex, low age-at-onset and A874V were independent factors associated with dysarthria. In addition, PTV, R778L, and P992L were predominant in early-onset patients, whereas A874V was predominant in late-onset patients, and patients with R778L/A874V genotype displayed a higher age-at-onset than patients with R778L/R778L or R778L/P992L genotype.

**Conclusions:**

Our work expanded the *ATP7B* variant spectrum and highlighted the differences among patients with WD in age-at-onset and *ATP7B* variants, which may provide some valuable insights into the diagnosis, counseling, and treatment of patients with WD.

**Supplementary Information:**

The online version contains supplementary material available at 10.1186/s40035-022-00287-0.

## Background

Wilson’s disease (WD, OMIM #277900) is an autosomal recessive disorder of copper metabolism caused by variants in the gene encoding a copper-transporting P-type ATPase (*ATP7B*) [1–3]. WD is characterized by hepatic or/and neurological symptoms, corneal Kayser–Fleischer’s (K–F) rings, low ceruloplasmin levels, and elevated 24-h urinary copper levels [4]. The clinically estimated prevalence of WD is 1/30,000 to 1/50,000 in the USA, Europe, and Asia, which is lower than the genetic prevalence due to factors such as above-zero onset age, shortened life expectancy, delayed diagnosis, overlooked cases, and low penetrance of some *ATP7B* variants [5, 6].

The canonical transcript of *ATP7B* (Ensembl ENST00000242839.10) contains 21 exons and encodes an eight-transmembrane-domain protein consisting of 1465 amino acids that is located on trans-Golgi networks (TGN). The protein is abundantly expressed in the liver and is expressed at lower levels in the kidneys, placenta, brain, lungs, and heart [2, 3, 7]. In the liver, ATP7B is responsible for copper incorporation into apoceruloplasmin and excretion of copper through bile; therefore, the inactivation of ATP7B leads to copper accumulation in the liver and subsequently in the brain, cornea and other tissues [8]. Currently, 782 pathogenic variants consisting of substitutions, deletions, insertions, and duplications have been identified in the *ATP7B* gene [9], and various effects such as altered intracellular localization, defective enzyme activity and reduced stability of the protein have been reported [10–13].

Patients with WD usually present with various phenotypes, with hepatic and/or neurological presentation being the main feature, and their ages of onset range from 8 months to 74 years [14–16]. The phenotype of WD is believed to be a comprehensive result of combinations of genetic, environmental and dietary factors, and several studies have reported that *ATP7B* truncating variants are associated with early onset of WD [17, 18]. So far, several common missense variants have been found all over the world, and a meta-analysis showed that H1069Q in Caucasians is associated with late and neurologic presentation of WD [19]. However, the effect of other common variants on the phenotype of WD is still elusive. In this study, we present a large cohort of patients with *ATP7B* variants in the aim to extend the variant spectrum and decipher the relationship between the phenotype and the genotypes of the most common variants in China, which are distinct from those in Caucasians.

## Methods

### Patients and data collection

**Fig. 1:**
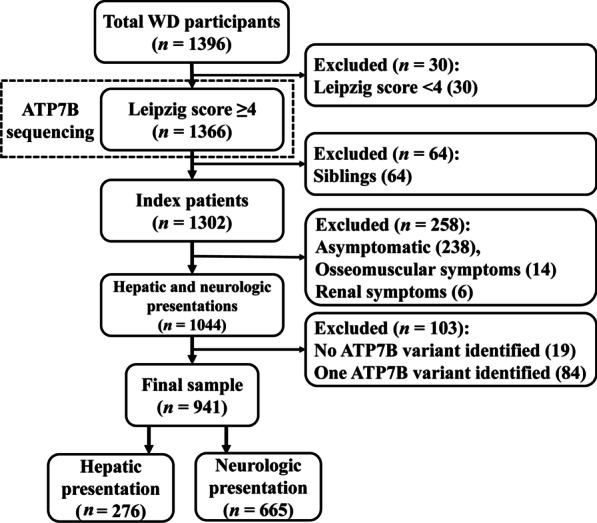
Flowchart of study participants

Consecutive patients who sought diagnosis and treatment for WD between August 31, 2016 and September 2, 2019 at the Department of Neurology of the First Affiliated Hospital of Anhui University of Chinese Medicine were recruited. Each patient was assessed by several neurologists, and their detailed clinical characteristics, including age at onset (the time of occurrence of initial symptoms attributable to WD), symptoms at presentation (distinguished as hepatic, neurologic, osteomuscular, renal, and asymptomatic subtypes), presence of K–F rings, age at diagnosis, and laboratory findings, were reviewed. Clinical diagnosis of WD was based on the Leipzig score [8, 20], and only patients who had a Leipzig score ≥ 4 were included for *ATP7B* variant analysis (Fig. 1).

### Variant analysis

Genomic DNA was extracted from the peripheral blood leukocytes of the patients using a standard protocol. For the index patients, exonic sequences and the intron–exon boundaries of *ATP7B* were amplified with the primers (Additional file 1: Table S1) and sequenced using an ABI3730xl DNA Analyzer (Applied Biosystems, Carlsbad, CA) following an order of exon 8, exon 13, exon 10_12, exon 18_19, exon 16, exon 5, exon 3, exon 15, exon 17, exon 2, exon 14, exon 20, exon 4, exon 7, exon 9, exon 6, exon 21, and exon 1 until at least two pathogenic variants were identified, or all of the 21 exons were sequenced. For patients identified with novel or ambiguous variants, all of the 21 exons were sequenced. Besides, the pathogenic variants in the probands were confirmed in their siblings with sequencing.

The variants identified in this study were compared with the list of reported pathogenic variations in the Human Gene Mutation Database (HGMD, accessed 18 August, 2021, http://www.hgmd.cf.ac.uk/ac/index.php) [21], the Wilson Disease Mutation Database (accessed 20 August, 2021, http://www.wilsondisease.tk/) [9], and the Ensembl database (accessed 20 August, 2021, https://asia.ensembl.org/index.html). The pathogenicity of the variants was identified with InterVar (http://wintervar.wglab.org/) or artificially by applying the American College of Medical Genetics and Genomics (ACMG) Standards and Guidelines [22, 23], and all the missense variants were assessed with SIFT (http://provean.jcvi.org/index.php) and PolyPhen2 (http://genetics.bwh.harvard.edu/pph2/index.shtml) [24, 25].

### Phenotype definition and genotype–phenotype assessment

The age and the symptoms at onset were used as markers of phenotype of WD as suggested previously [8, 26]. The patients presenting with active clinical hepatic symptoms (jaundice, anorexia, nausea, coagulopathy, ascites, etc.) were classified as having a hepatic subtype, and the patients who presented with neurological features (dystonia, tremor, gait abnormality, swallowing difficulty, dysarthria, salivation, mental illness, etc.) with or without hepatic features were classified as having a neurological subtype [8]. The patients identified incidentally during physical examinations were classified as having an asymptomatic subtype, as they could develop either symptomatic hepatic or neurologic disease [26]. A small proportion of patients who had arthralgia, arthritis or renal symptoms at onset were classified as ‘Others’, and the siblings were analyzed separately. Index patients with the hepatic phenotype were further divided into groups with acute liver disease and chronic liver disease as recommended in a previous study [20], and those with the neurological phenotype were further analyzed in groups with predominant clinical signs of dystonia, gait abnormality, swallowing difficulty, dysarthria, tremor, and salivation. The genotype–phenotype assessment was performed within index patients presenting with liver or neurologic diseases, and only patients who were identified with two potential pathogenic *ATP7B* variants were included (Fig. 1).

### Statistical analysis

All statistical analyses were performed using SPSS version 23 (IBM, Armonk, NY). Quantitative data (age at onset and diagnosis) are expressed as mean ± SD, and categorical variables are given as absolute (number) and relative frequencies (%). To compare continuous variables, Student’s *t*-test was used for 2-group comparison, and one-way analysis of variance followed by Scheffe multiple comparison test was used for comparison of 3 or more groups, as appropriate. For categorical variables, the *χ*^2^ test or Fisher’s exact test was used, as appropriate. Binary logistic regressions were performed to identify the factors (sex, age at onset, and presence of protein-truncating variants [PTV] or hotspot variants R778L, P992L, and A874V [dominant model]) associated with neurologic/hepatic presentation, acute/chronic hepatic disease, or specific neurological symptom (dystonia, tremor, gait abnormality, swallowing difficulty, dysarthria, or salivation). Only factors that showed significant associations in univariate analyses were included in the multivariate analysis. To control type I errors caused by multiple-hypothesis test, the *P*-value was adjusted by the Benjamini–Hochberg method [27]. The criterion for a significant difference was *P* < 0.05.

## Results

### Demographic features of the patients

Based on the inclusion and exclusion criteria, 1366 patients (1302 index patients and 64 siblings) were enrolled for *ATP7B* sequencing (Fig. 1). Of the 1302 index patients, 307 (23.58%) presented with hepatic symptoms, 737 (56.61%) presented with neurologic symptoms, 15 (1.15%) presented with osseomuscular symptoms, and 5 (0.38%) presented with renal symptoms. The remaining 238 (18.28%) patients were diagnosed with elevated transaminases or K–F rings, and were categorized as asymptomatic subtype. Of the 64 siblings, a majority of them were asymptomatic and the others presented with hepatic, neurologic, or osseomuscular symptoms. The demographic characteristics are summarized in Table 1.

**Table 1:** Diagnostic findings in patients with WD (*n* = 1366)

	Hepatic (*n* = 307)	Neurologic (*n* = 737)	Asymptomatic (*n* = 238)	Others (*n* = 20)	Siblings (*n* = 64)
Males (%)	173 (56.4)	437 (59.3)	161 (67.6)	12 (60.0)	38 (59.4)
Age at onset (years)					
Range	2.6–64.8	5.5–62.6	1.8–50.9	4.7–26.0	-
Mean ± SD	17.6 ± 10.4	19.3 ± 8.0	9.5 ± 8.0	12.0 ± 5.5	-
Age at diagnosis (years)					
Range	3.1–65.6	5.5–62.8	2.0–54.2	5.3–26.8	2.8–48.0
Mean ± SD	18.5 ± 10.8	20.2 ± 8.4	10.4 ± 8.7	13.2 ± 5.1	13.8 ± 10.9
Presented symptoms (male/female)	Acute hepatic WD (39/35) Chronic hepatic WD (134/99)	Dystonia (289/193) Tremor (325/200) Gait abnormality (204/153) Dysarthria (298/228) Swallowing difficulty (88/55) Salivation (166/125)	Elevated transaminases (160/73) K–F rings (1/4)	Osseomuscular (8/7) Renal (4/1)	Asymptomatic (29/24) Hepatic (3/0) Neurologic (5/2) Renal (1/0)
CPL < 0.1 g/l (%)	275/303 (90.8)	698/728 (95.9)	208/227 (91.6)	19/20 (95.0)	58/60 (96.7)
K–F ring (%)	273 (88.9)	708 (96.1)	117 (49.2)	17/3 (85.0)	36 (56.3)

*SD* standard deviation, *CPL* ceruloplasmin level, *K–F rings* Kayser–Fleischer rings

### Characterization of genetic variants in the *ATP7B* gene

**Fig. 2:**
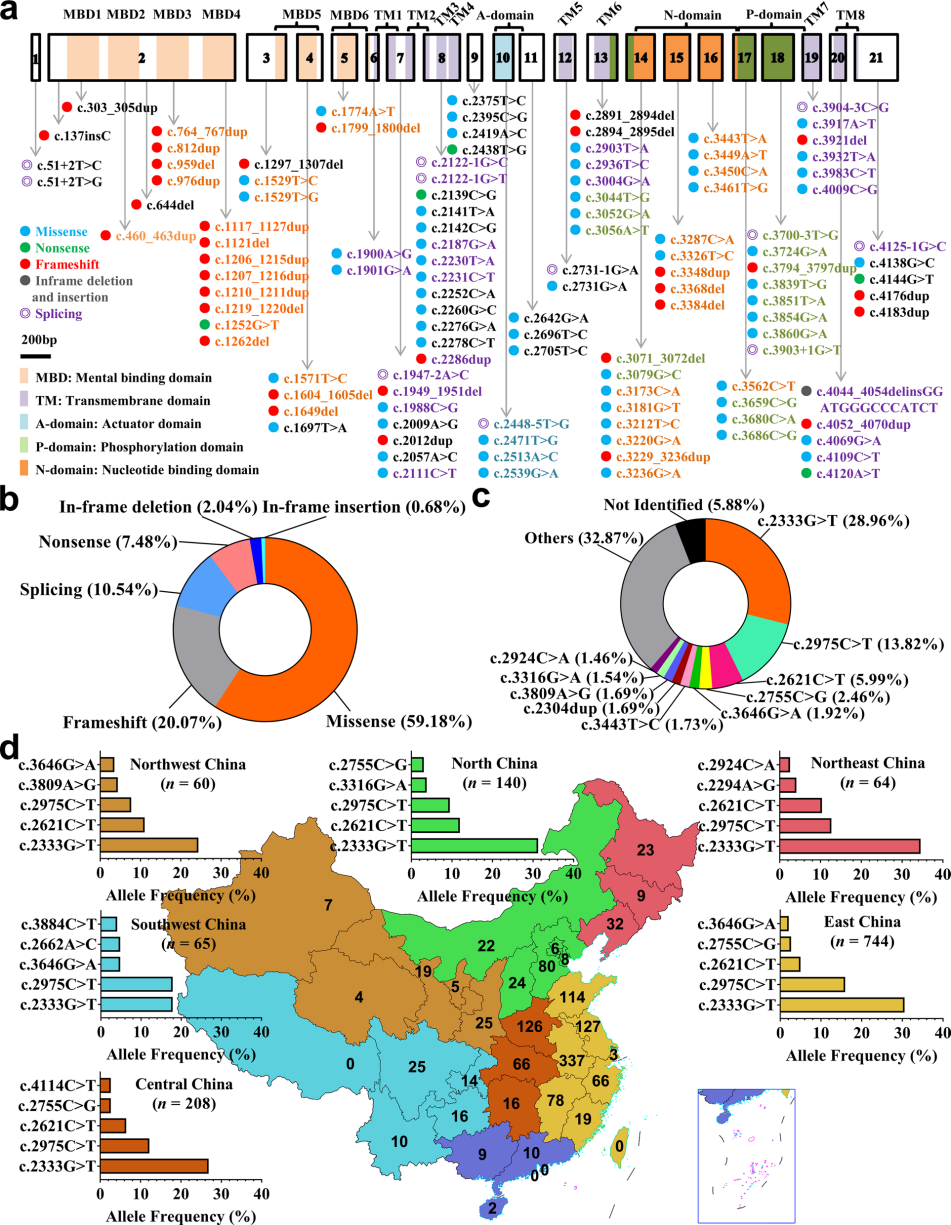
Characterization of genetic variants in the *ATP7B* gene. **a** Novel variants were visualized in relation to the ATP7B protein regions. **b** Various mutants identified in this study and their proportions. **c** Allele frequency of common *ATP7B* variants in Chinese patients. **d** Family distributions of patients in this study and allele frequencies of common variants in different regions of China

Collectively, 294 underlying pathogenic variants (Additional file 2: Table S2), excluding synonymous variants, SNPs with high allele frequencies, and previously mentioned nonpathogenic variants (Additional file 3: Table S3), were identified in the 1302 index patients, among which 116 were novel (Fig. 2a). The variants consisted of 174 (59.18%) missense variants, 59 (20.07%) frameshift variants, 31 (10.54%) splice-site variants, 22 (7.48%) nonsense variants, 6 (2.04%) in-frame deletions and 2 (0.68%) in-frame insertions (Fig. 2b). Variants were distributed throughout exons of *ATP7B*, with a majority of variants before mental binding domain 5 (MBD5) being PTV (Additional file 2: Table S2). The most common variant was c.2333G>T (R778L, exon 8), accounting for 28.96% (754/2604) of the total alleles, followed by c.2975C>T (P992L, exon 13, 13.82%), c.2621C>T (A874V, exon 11, 5.99%), c.2755C>G (R919G, exon 12, 2.46%), and c.3646G>A (V1216M, exon 17, 1.92%) (Fig. 2c). In this cohort, the 1302 index patients came from 30 different provinces in China, mostly from Anhui (337, 25.88%), Jiangsu (127, 9.75%), Henan (126, 9.68%), and Shandong (114, 8.76%) Provinces, and the hotspot variants varied slightly by geographic regions (Fig. 2d).

The 116 novel variants included 64 missense variants (Additional file 4: Fig. S1), 35 small insertions and deletions (Additional file 4: Fig. S2a), 12 splice-site variants (Additional file 4: Fig. S2b), and 5 nonsense variants (Additional file 4: Fig. S2c). According to the ACMG Standards and Guidelines, the 5 nonsense variants, 34 of the 35 small insertion and deletion variants, and 9 canonical ± 1 or 2 splice-site variants resulting in defective ATP7B proteins could be considered as ‘pathogenic variants’. The shift variant p.1348_1352delins MDGPIS (c.4044_4054delinsGGATGGGCCCATCT) resulting in in-frame insertion could be classified as ‘likely pathogenic variants’. The other three splice-site variants (2448-5T>G, c.3700-3T>G, c.3904-3C>G), which needed further functional analysis, were classified as ‘variants with uncertain significance’ at this stage. The 64 missense variants were all located in highly conserved regions of ATP7B (Additional file 4: Fig. S3), and results from SIFT, PolyPhen-2, and InterVar analysis (Additional file 2: Table S2) indicated that all of them could be classified as ‘likely pathogenic variants’.

Overall, among the 1302 unrelated patients clinically diagnosed with WD, 1167 patients (89.63%) were characterized with two potential disease-causing variants (204 homozygotes and 963 compound heterozygotes), 115 patients (8.83%) were characterized with one potential disease-causing variant, and 20 patients (1.54%) were characterized with no potential disease-causing variants (Table 2). There were 532 different variant combinations identified (Additional file 5: Table S4), among which the most common genotype was R778L/R778L, accounting for 9.60% (125/1302) of the index patients, followed by R778L/P992L (6.91%), R778L/A874V (4.07%), P992L/P992L (3.00%), and R778L/R919G (1.92%) (Table 2).

**Table 2:** Genotypes of the index patients (*n* = 1302)

Genotype	Index patients	Hepatic	Neurologic	Asymptomatic	Others
Total	1302 (100%)	307 (100%)	737 (100%)	238 (100%)	20 (100%)
Homozygotes	204 (15.67)	44 (14.33)	121 (16.42)	37 (15.55)	2 (10.00)
R778L/R778L	125 (9.60)	24 (7.82)	74 (10.04)	26 (10.92)	1 (5.00)
P992L/P992L	39 (3.00)	7 (2.28)	27 (3.66)	4 (1.68)	1 (5.00)
A874V/A874V	6 (0.46)	2 (0.65)	2 (0.27)	2 (0.84)	0
PTV/PTV	10 (0.77)	1 (0.33)	9 (1.22)	0	0
Others	24 (1.84)	10 (3.26)	18 (2.44)	5 (2.10)	0
Heterozygotes	963 (73.96)	232 (75.57)	544 (73.81)	172 (72.27)	15 (75.00)
PTV/PTV	24 (1.84)	9 (2.93)	12 (1.63)	3 (1.26)	0
PAV/PAV	645 (49.54)	149 (48.53)	368 (49.93)	118 (46.58)	10 (50.0)
R778L/P992L	90 (6.91)	16 (5.21)	54 (7.33)	18 (7.56)	2 (10.00)
R778L/A874V	53 (4.07)	7 (2.28)	39 (5.29)	6 (2.52)	1 (5.00)
R778L/R919G	25 (1.92)	4 (1.30)	16 (2.17)	5 (2.10)	0
R778L/S975Y	16 (1.23)	2 (0.65)	11 (1.49)	2 (0.84)	1 (5.00)
P992L/A874V	16 (1.23)	3 (0.98)	11 (1.49)	2 (0.84)	0
R778L/V1106I	13 (1.00)	7 (2.28)	3 (0.41)	3 (1.26)	0
R778L/V1216M	12 (0.92)	0	9 (1.22)	3 (1.26)	0
Others	420 (32.26)	110 (35.83)	225 (30.53)	79 (33.19)	6 (30.00)
PAV/PTV	294 (22.58)	74 (24.10)	164 (22.25)	51 (21.43)	5 (25.00)
R778L/PTV	96 (7.37)	25 (8.14)	58 (7.87)	12 (5.04)	1 (5.00)
P992L/PTV	48 (3.69)	12 (3.91)	24 (3.26)	12 (5.04)	0
A874V/PTV	24 (1.84)	1 (0.33)	18 (2.44)	4 (1.68)	1 (5.00)
R919G/PTV	17 (1.31)	4 (1.30)	9 (1.22)	4 (1.68)	0
Others	109 (8.37)	32 (10.42)	55 (7.46)	19 (7.98)	3 (15.00)
One variant identified	115 (8.76)	23 (7.49)	61 (8.28)	28 (11.76)	3 (15.00)
R778L/?	33 (2.53)	10 (3.26)	19 (2.58)	4 (1.68)	0
P992L/?	29 (2.23)	4 (1.30)	13 (1.76)	10 (4.20)	2 (10.00)
PTV/?	17 (1.31)	1 (0.33)	13 (1.76)	2 (0.84)	0
A874V/?	10 (0.77)	3 (0.98)	5 (0.68)	2 (0.84)	0
Other/?	26 (2.00)	5 (1.63)	11 (1.49)	10 (4.20)	1 (5.00)
No variant identified	20 (1.54)	8 (2.61)	11 (1.49)	1 (0.42)	0

PTV, protein-truncating variants (e.g., frameshift, nonsense, splice sites); PAV, protein-altering variants (e.g., missense, in-frame deletions and insertions); ? second variants unknown. The data are presented as number and percentage of patients in each group

### Association of sex, age at onset, and *ATP7B* variants with WD manifestations

Next, we focused our attention on index patients who carry two potential pathogenic *ATP7B* variants and had onset with hepatic (*n* = 276) or neurologic (*n* = 665) symptoms (Fig. 1). We noticed that the male patients were dominant in both hepatic (male/female: 152/124) and neurologic groups (male/female: 396/269, *P* = 0.205), which was different from the previous finding in Caucasians that females were more common in the hepatic group [26]. In addition, in patients incidentally identified based on elevated transaminase levels at an average age, there was a male predominance (male/female: 160/73) compared to those with a hepatic presentation (male/female: 152/124, *P* = 0.002) and those with a neurologic presentation (male/female: 396/269, *P* = 0.014), implying that liver injury tends to start at an earlier age in males than in females. In fact, males had an earlier age at onset than females in the hepatic group (males *vs* females: 15.71 ± 9.15 years *vs* 19.28 ± 11.39 years, *P* = 0.004), but not in the neurologic group (males *vs* females: 19.57 ± 8.66 years *vs* 18.89 ± 7.49 years, *P* = 0.298).

**Fig. 3:**
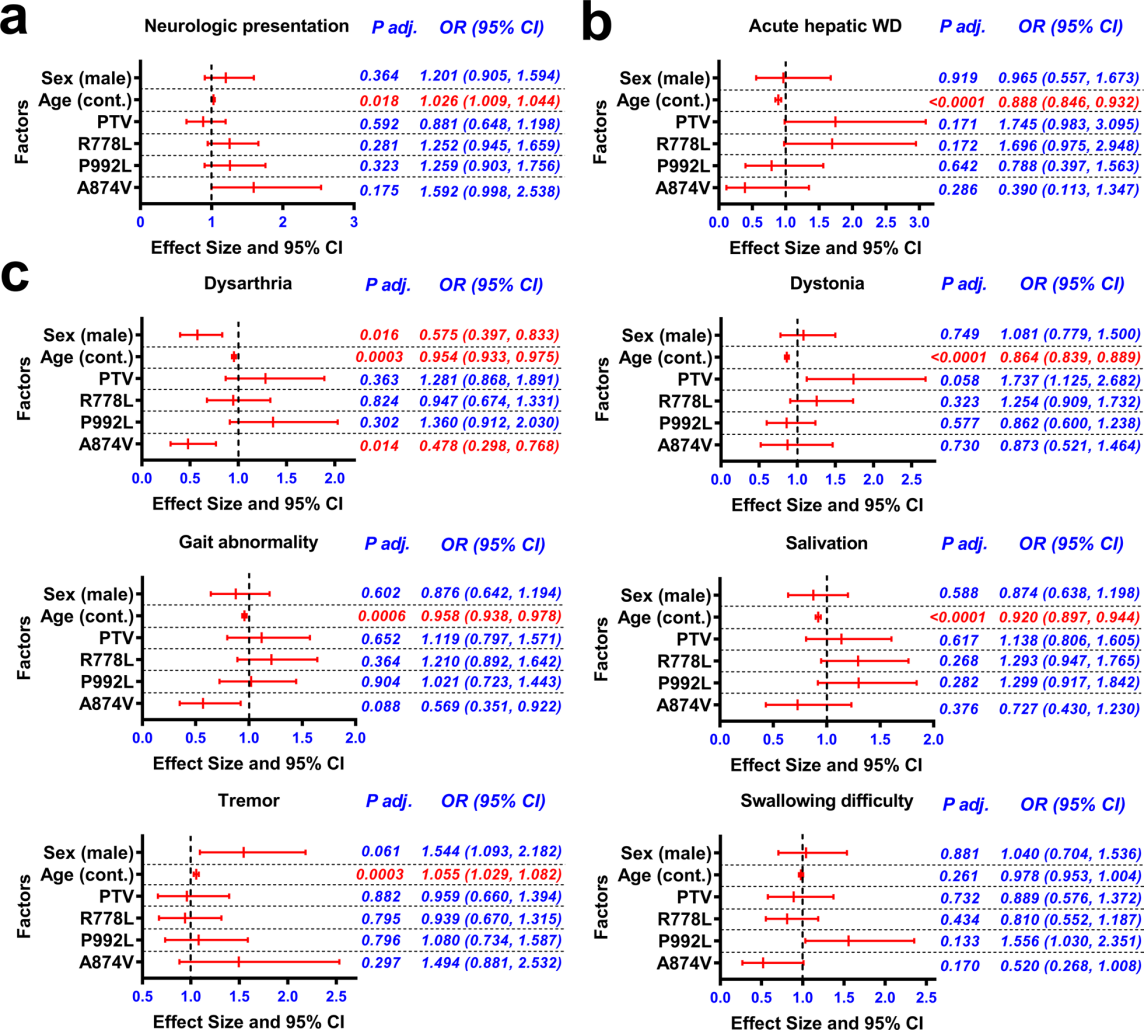
Associations of sex (male), age-at-onset, and *ATP7B* variants with WD manifestations. **a** Binary logistic regression analysis of factors associated with neurologic presentation in hepatic and neurologic presentation patients (*n* = 941). **b** Binary logistic regression analysis of factors associated with acute hepatic WD in hepatic presentation patients (*n* = 276). **c** Binary logistic regression analysis of factors associated with dystonia, gait abnormality, swallowing difficulty, dysarthria, tremor, or salivation in neurologic presentation patients (*n* = 665). Only factors with significant associations in univariate analyses were included in the multivariate analysis. The *P*-values were adjusted with the Benjamini–Hochberg method. Cont., continuous variable, PTV, protein-truncating variants (e.g., frameshift, nonsense, splice sites)

Binary logistic regression confirmed that there was no significant correlation between sex (male) and neurological presentation, and no association between the presence of PTV or the three common variants (R778L, P992L, and A874V) and neurological presentation (Fig. 3a). Instead, we did find that the age-at-onset, which represents the natural progression time of the disease, was a factor related to neurologic presentation (Fig. 3a), consistent with the fact that neurologic symptoms appeared later than hepatic symptoms (hepatic *vs* neurologic: 17.32 ± 10.36 years *vs* 19.17 ± 7.99 years, *P* = 0.008). Further analysis was performed in the hepatic or neurologic presentation groups with various symptoms, and the results indicated that the low age-at-onset was a factor associated with acute hepatic disease in patients with hepatic presentation (Fig. 3b), and was a factor associated with dystonia, gait abnormality, and salivation in patients with neurological presentation (Fig. 3c). Besides, high age-at-onset was a factor associated with tremor, while sex (male), low age-at-onset and A874V variant were independent factors associated with dysarthria in patients with neurological presentation (Fig. 3c).

### Genotype–phenotype correlation of hotspot variant combinations

Further analysis was focused on common homozygotes and compound heterozygotes in the hepatic and neurologic groups. As shown in Table 2, there was no significant difference in the distribution of R778L/R778L, P992L/P992L, R778L/PTV, PTV/PTV, R778L/P992L, and R778L/A874V genotypes in hepatic and neurologic groups, implying no association of the hotspot variant combinations with hepatic or neurologic presentation.

**Fig. 4:**
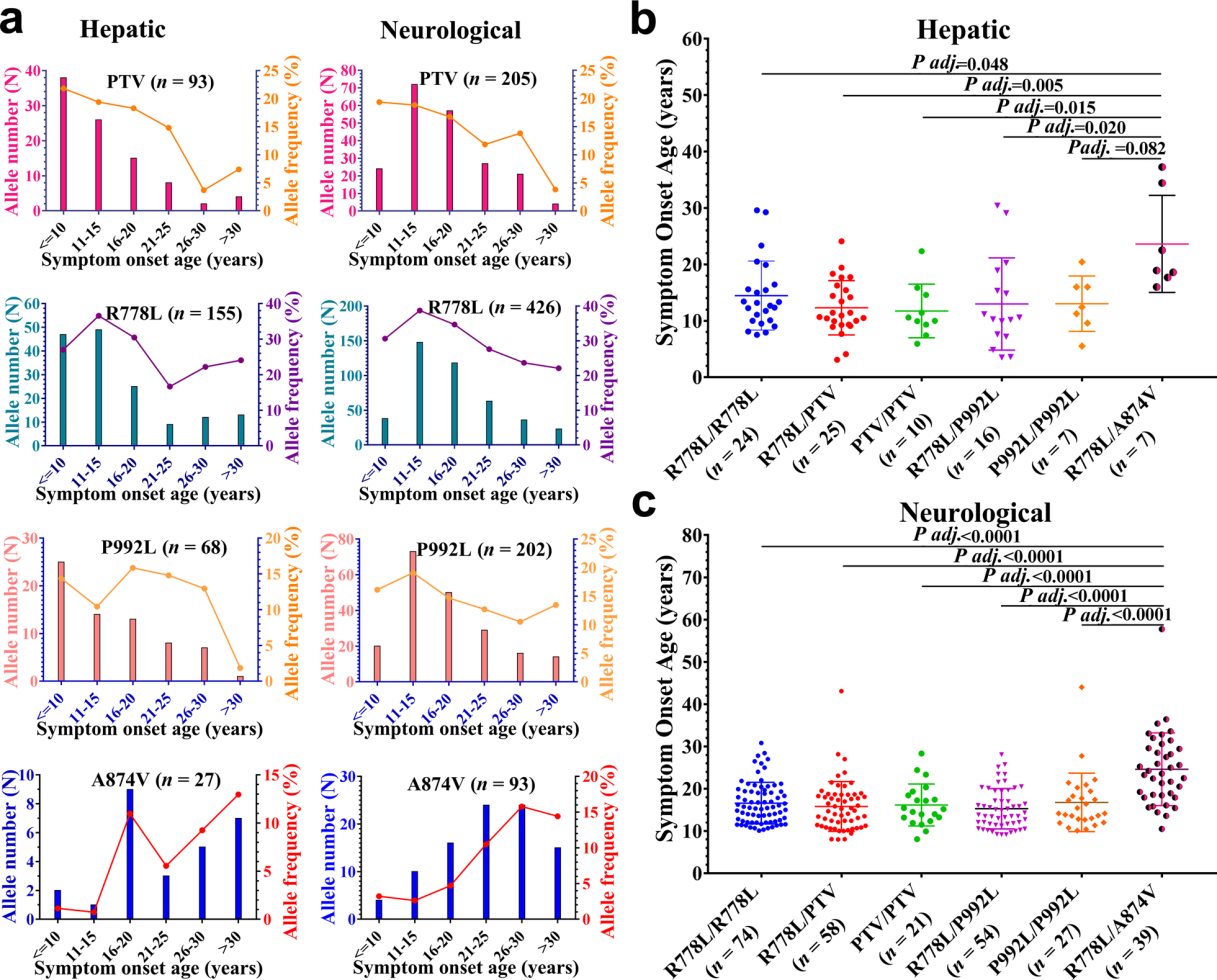
Effect of common *ATP7B* genotypes on symptom onset age of WD. **a** Allele frequencies of target variants according to age at onset. **b** Effects of genotype on age-at-onset in patients with hepatic presentation. **c** Effects of genotype on age-at-onset in patients with neurologic presentation. Data were evaluated by one-way analysis of variance followed by Scheffe multiple comparison test. PTV, protein-truncated variants (e.g., frameshift, nonsense, splice sites)

Next, we evaluated the distribution frequencies of several common variants (PTV, R778L, P992L, and A874V) and their percentages in specific ranges of age-at-onset. As shown in Fig. 4a, PTV, R778L, and P992L were predominant in early-onset patients, whereas A874V was predominant in late-onset patients regardless of the hepatic or neurologic group. Interestingly, patients with R778L/R778L and P992L/P992L homozygotes, as well as patients with R778L/PTV, PTV/PTV, and R778L/P992L compound heterozygotes, had comparable age-at-onset, either in the hepatic or in the neurologic group, and patients with the R778L/A874V genotype displayed a higher age-at-onset in both the hepatic and the neurologic groups, compared to patients with R778L/R778L or R778L/P992L genotype (Fig. 4b, c).

## Discussion

In this study, we  performed targeted sequencing of *ATP7B* in 1302 index patients from 30 provinces of China, which allowed us to delineate the variant spectrum, clinical features, and genotype–phenotype correlations of WD in China. Overall, 294 potential pathogenic variants were identified in this study, among which 178 have been reported to be disease-causing variants in the Wilson Disease Mutation Database [9]. The remaining 116 variants were novel, and the current evidence indicated that 48 of them could be classified as ‘pathogenic variants’, 65 of them could be classified as ‘likely pathogenic variants’, and 3 of them could be classified as ‘variants with uncertain significance’. These findings substantially expand the known spectrum of pathogenic *ATP7B* variants. Significantly, two of the previously identified variants (I390V and A476T), which were predicted to be ‘tolerated’ and ‘benign’, and two of the novel variants (L510R and L1154R), which were predicted to be ‘damaging’ and ‘probably damaging’, were found to co-exist with two other known pathogenic variants in the patients (Additional file 5: Table S4). Their roles should be further analyzed.

The top five most common variants were R778L, P992L, A874V, R919G, and V1216M in our cohort, and there were slight differences based on geographical region. As most of our patients came from Anhui, Jiangsu, Henan, and Shandong Provinces, the imbalanced distribution of patients and common variants in different geographical areas of China may contribute to the discrepancies in variant “hotspots” between our study and previous studies in China, where the majority of patients came from Fujian and Zhejiang Provinces [28, 29]. In total, 532 different variant combinations were identified in 1167 patients, which seems to be more complicated than that in Caucasians [26]. The overall genetic diagnostic rate was 89.63% (1167/1302), which was comparable to previous results obtained by exon-by-exon sequencing (919/1172, 78.4%) in Caucasians, whole-exome sequencing (218/248, 87.9%) in Poland, and whole sequencing of the 5’UTR, 21 exons and their flanking regions (569/632, 90.0%) in China [26, 28, 30]. Several previously mentioned factors, such as large hemizygous deletions, regulatory region variants, and genetic alterations outside *ATP7B* may contribute to the fact that not all patients are genetically diagnosed [26, 28].

We noticed that patients with neurologic symptoms were predominant in our cohort, which was different from previous studies in Europe and Korea [26, 31]. There may be a screening effect of our institution; however, the predominance of neurological patients has also been noted in another cohort from China [32]. Meanwhile, we noticed a predominance of male patients in different WD groups compared to the sex distribution in the general population (males/females: 105.07/100, according to the 2020 Population Census of China), which was consistent with the results of several other studies in Asia [31–33]. The predominance of male patients in the neurologic group was also noticed in Caucasians [26, 34], and was plausibly explained by the protective role of estrogens in the brain [30]. However, the opposite phenomenon was observed in Caucasians with hepatic symptoms [26, 34], which may be a result of the different *ATP7B* variant spectrum in our study. In the current study, the proportion of male patients was significantly higher in the group diagnosed based on elevated transaminase levels than in the groups of patients diagnosed based on hepatic or neurologic symptoms. Considering that patients with elevated transaminase levels were primarily identified during preschool physical examinations at an average age, we speculated that hepatic injury may start at an earlier age in males than in females, which was found to be statistically significant in patients with hepatic presentation. Besides, it has been reported that the penetrance of some pathogenic variants in WD may be less than 100% [5, 6]; a sex-dependent penetrance and unequal concerns for male and female patients should also be considered.

As previously recommended, the age and symptoms at onset were used as phenotype descriptors in this study [26]. A key factor in finding genotype–phenotype correlation should be timely and definite diagnosis of symptoms and age at onset in patients with WD. However, there is a delay of diagnosis, mild hepatic or neurological symptoms may be overlooked by a physician, and the boundary between neurological and hepatic presentations is not always clear. In this study, the phenotypic analysis was performed in patients with active clinical hepatic and neurological presentation. The asymptomatic patients exhibiting elevated transaminases were excluded as they may progress to active clinical hepatic or neurological symptoms later without timely diagnosis and treatment. The result indicated that PTV, R778L, P992L, and A874V were not associated with neurological presentation, but the high age-at-onset played a role. This is consistent with the presumed natural history of WD, in which copper accumulates first in the liver and then extrahepatic tissues when the hepatic copper storage capacity is exceeded [26]. We also observed that the low age-at-onset was associated with acute hepatic disease and some specific neurological symptoms. This may be a result from different processes involved in copper-induced hepatocyte apoptosis or oxidative stress in the liver [4, 35], and distinct susceptibility to copper toxicity in brain regions at different developmental stages. However, we did observe an association between A874V and dysarthria. Since the ATP7B protein is also expressed in different regions of the brain [36], the properties of various ATP7B variants may play a role in the occurrence of dysarthria. Interestingly, inhibition of the p38/JNK pathway involved in degradation of the H1069Q or R778L mutant failed to rescue the A874V mutant, implying distinct property and metabolic pathway of A874V variant [13].

In addition, we assessed the allele frequency of PTV according to age-at-onset, and found that PTV was more common in patients with a younger age-at-onset in both the hepatic and neurologic groups, which was consistent with the previous finding that truncating variants are associated with an early onset of WD [17, 18]. Based on the same criterion, we performed a frequency assessment of the variants R778L, P992L and A874V, and found that the R778L and P992L variants were enriched in earlier-onset WD, whereas the A874V variant was enriched in later-onset WD. The results were confirmed by comparing patients with the specific genotypes of R778L/R778L, P992L/P992L, R778L/PTV, PTV/PTV, R778L/P992L and R778L/A874V in both the hepatic and the neurologic groups, which revealed that patients with the R778L/A874V genotype tended to have a later onset than patients with R778L/R778L or R778L/P992L genotype. This implies that the role of a specific variant may vary in different variant combinations.

The different presentations of WD may result from various properties associated with the *ATP7B* variant, such as the subcellular localization, stability, catalytic activity and copper transport activity. In hepatocytes, normal ATP7B protein is localized in the TGN, whereas the truncated ATP7B shows a diffuse, clustered, cytoplasmic pattern of localization that is distinct from the pattern of localization in the TGN or endoplasmic reticulum (ER) [37]. The missense variants display various localization patterns, with R778L and A874V predominantly located in ER, and H1069Q located in both the ER and TGN [11, 12]. The phosphorylation activity is also altered, with A874V associated with significantly increased ATP7B phosphorylation and H1069Q associated with defective ATP-binding ability [11]. In addition, the copper transport activities of the A874V, P992L and H1069Q variants are reduced to varying degrees [11]. In *Saccharomyces cerevisiae*, the *ccc2* mutant can be partially rescued by H1069-ATP7B, whereas only weak complementation was found with R778L-ATP7B and P992L-ATP7B [38, 39]. Recently, a copper gradient maintained by vesicular ATP7B in mouse intestine has been reported [40], and the effects of different variants on the buffering ability of ATP7B should also be considered.

## Conclusions

In summary, our study identified 116 novel variants that substantially expand the spectrum of pathogenic *ATP7B* variants, and demonstrated that PTV, R778L, P992L and A874V were not associated with hepatic or neurologic presentation. Besides, we also noted no correlations of PTV, R778L, P992L and A874V with acute hepatic disease, dystonia, gait abnormality, salivation, tremor and swallowing difficulty, except that A874V was negatively associated with dysarthria. In addition, patients with the R778L/A874V genotype displayed a higher age-at-onset than patients with R778L/R778L or R778L/P992L genotype. Our research highlights the differences among WD patients in age-at-onset and *ATP7B* variants, which may provide some valuable insights into the diagnosis, counseling, and treatment of patients with WD. Comparing the clinical features of patients with R778L/R778L and R778L/A874V genotypes and studying their underlying mechanisms will be helpful for understanding the progression of WD.

## Supplementary Information

**Additional file 1: Table S1.** Primers used for amplification and sequencing of exons and exon–intron boundaries of *ATP7B*.

**Additional file 2: Table S2.** Details of potential pathogenic variants of *ATP7B* identified in this study.

**Additional file 3: Table S3.** Non-pathogenic variants of *ATP7B* identified in this study.

**Additional file 4: Fig. S1.** Sequencing results for 64 novel missense variants of the *ATP7B* gene. **Fig. S2.** Sequencing results for novel variants of the *ATP7B* gene except for missense variants. **Fig. S3.** Homology comparisons of novel missense variants in the ATP7B protein.

**Additional file 5: Table S4.** *ATP7B* variant combinations in this cohort.

## Data Availability

The data presented in the current study are available on request from the corresponding authors.

## Abbreviations

WD: Wilson’s disease
K–F: Kayser–Fleischer
TGN: Trans-Golgi networks
HGMD: The Human Gene Mutation Database
ACMG: The American College of Medical Genetics and Genomics
PTV: Protein-truncating variants
PAV: Protein-altering variants
ER: Endoplasmic reticulum
