# Supplementary material for: Clinical and genetic characterization of a large cohort of patients with Wilson’s disease in China
Source: Transl Neurodegener. 2022 Feb 28;11:13. doi: 10.1186/s40035-022-00287-0 (PMC8883683; doi:10.1186/s40035-022-00287-0)
Supplement: Supplementary file 1 — Additional file 1: Table S1. Primers used for amplification and sequencing of exons and exon–intron boundaries of ATP7B. [file 40035_2022_287_MOESM1_ESM.docx]

Table S1. Primers used for amplification and sequencing of exons and exon-intron boundaries of *ATP7B*.

| **Names** | **Sequences (5’-3’)** | **Length** |  |
| --- | --- | --- | --- |
| Exon1-F | CACTCTCCCCTCACGCTCTCAT | 533bp |  |
| Exon1-R | GCGGGGTTTTCCATTGCTCC |  |  |
| Exon2-F | ACTAAGGAGGACAGACAGTT | 1607 bp |  |
| Exon2-R | TAACATGCAAGGAAAGTTTG |  |  |
| Exon2-S1 | AGAGAAGCTGGGATGTTGTA |  |  |
| Exon2-S2 | TTATCTCATTCAGCCCGAAG |  |  |
| Exon3-F | ACAATGAACCCTCACCAAGA | 506bp |  |
| Exon3-R | TACAAGGACATTAGACAAAC |  |  |
| Exon4-F | AAACAGTGTTTGAAAACTGC | 350bp |  |
| Exon4-R | TCCAAAATGCAAACTGTCAG |  |  |
| Exon5-F | GAATCTCACATGCGGGGTTT | 449bp |  |
| Exon5-R | CCATGGGAAAAGTTGAAGAA |  |  |
| Exon6-F | ATGAAGATTTCTGACCATTAGG | 421bp |  |
| Exon6-R | TTAGATGAGAGCTGGAGTTT |  |  |
| Exon7-F | GCAGGTCTTAAACTGTGTCC | 330bp |  |
| Exon7-R | AATATCTGAGGGCCACACAC |  |  |
| Exon8-F | ATAAACGCCCATCACAGAGG | 524bp |  |
| Exon8-R | GCACCTTAATTATATGGAGGTTTCC |  |  |
| Exon9-F | GTTTCTCTCGCACCAGCTGT | 258bp |  |
| Exon9-R | TGCAGCTCACACAGATTGAT |  |  |
| Exon10_12-F | CCTAGAACCTGACCCGGTGA | 830bp |  |
| Exon10_12-R | CACCATATAGCCCAAGGCAT |  |  |
| Exon13-F | GCCCCCCTGAAATGTCCTTA | 371 bp |  |
| Exon13-R | TGGCTCTCAGGCTTTTCTCT |  |  |
| Exon14-F | ACACATAGGGAAACAGTTTC | 435bp |  |
| Exon14-R | GTGAGGAATAAAAGAGCATT |  |  |
| Exon15-F | ACCTCCCTCCCCTCCTTTCT | 587 bp |  |
| Exon15-R | GAAGCAAGACCGATATGGGA |  |  |
| Exon16-F | CACAAGAGGTGCTTACAAGG | 387bp |  |
| Exon16-R | GAAGGCTTTTGTTTGTCTTC |  |  |
| Exon17-F | CAATTATATTGCTTCCAGAC | 376 bp |  |
| Exon17-R | CTTTACACAGTTTGCAACAT |  |  |
| Exon18_19-F | GGCCTAAACCAGTGCAGGGT | 680bp |  |
| Exon18_19-R | TCACTAACCCCAGCAGGAAC |  |  |
| Exon20-F | ACACCCAGCTTTCTAGGAAG | 367bp |  |
| Exon20-R | TCCACTGTGCTAAGCATGCA |  |  |
| Exon21-F | AATGGCTCAGATGCTGTTGC | 361bp |  |
| Exon21-R | GCTTGTGGTGAGTGGAGGCA |  | |
