# Supplementary material for: Clinical and genetic characterization of a large cohort of patients with Wilson’s disease in China
Source: Transl Neurodegener. 2022 Feb 28;11:13. doi: 10.1186/s40035-022-00287-0 (PMC8883683; doi:10.1186/s40035-022-00287-0)
Supplement: Supplementary file 2 — Additional file 2: Table S2. Details of potential pathogenic variants of ATP7B identified in this study. [file 40035_2022_287_MOESM2_ESM.docx]

Table S2. Details of possible pathogenic variants.

| Nucleotide variant | Protein alteration | Exon | Area of protein | Variation type | SIFT ^a^ | | PolyPhen2 ^b^ | | InterVar ^c^ | Class. | Freq. (%) | No.of patients | |
| --- | --- | --- | --- | --- | --- | --- | --- | --- | --- | --- | --- | --- | --- |
|  |  |  |  |  | **Score** | **Prediction** | **Score** | **Prediction** |  |  |  | **Hom.** | **Het.** |
| c.51+1G>A | n.a | 1 | Before MBD1 | Splicing | n.a | n.a | n.a | n.a | 1*PVS | P | 0.08 | 0 | 2 |
| c.51+2T>C | **n.a** | **1** | **Before MBD1** | **Splicing** | **n.a** | **n.a** | **n.a** | **n.a** | **1*PVS** | **P** | **0.04** | **0** | **1** |
| c.51+2T>G | **n.a** | **1** | **Before MBD1** | **Splicing** | **n.a** | **n.a** | **n.a** | **n.a** | **1*PVS** | **P** | **0.15** | **0** | **4** |
| c.122A>G | p.N41S | 2 | Before MBD1 | Missense | 0.014 | Damaging | 0.946 | Possibly damaging | 1*PM, 2*PM, 3*PP, 5*PP | LP | 0.15 | 0 | 4 |
| c.137ins | **p.G46AfsX31** | **2** | **Before MBD1** | **Frameshift** | **n.a** | **n.a** | **n.a** | **n.a** | **1*PVS** | **P** | **0.04** | **0** | **1** |
| c.254G>T | p.G85V | 2 | MBD1 | Missense | 0.001 | Damaging | 1.000 | Probably damaging | 3*PS, 2*PM, 3*PP, 5*PP | P | 0.04 | 0 | 1 |
| c.268_270del | p.K90del | 2 | MBD1 | Inframe del | n.a | n.a | n.a | n.a | 4*PM | LP | 0.04 | 0 | 1 |
| c.303_305dup | **p.Y102X** | **2** | **MBD1** | **Frameshift** | **n.a** | **n.a** | **n.a** | **n.a** | **1*PVS** | **P** | **0.04** | **0** | **1** |
| c.314C>A | p.S105X | 2 | MBD1 | Nonsense | n.a | n.a | n.a | n.a | 1*PVS, 2*PM, 3*PP, 5*PP | P | 0.12 | 0 | 3 |
| c.460_463dup | **p.Q155LfsX8** | **2** | **MBD2** | **Frameshift** | **n.a** | **n.a** | **n.a** | **n.a** | **1*PVS** | **P** | **0.04** | **0** | **1** |
| c.525dup | p.V176SfsX28 | 2 | MBD2 | Frameshift | n.a | n.a | n.a | n.a | 1*PVS | P | 0.73 | 0 | 19 |
| c.571_575del | p.L190SfsX10 | 2 | MBD2 | Frameshift | n.a | n.a | n.a | n.a | 1*PVS | P | 0.04 | 0 | 1 |
| c.644del | **p.A215VfsX2** | **2** | **MBD2/MBD3** | **Frameshift** | **n.a** | **n.a** | **n.a** | **n.a** | **1*PVS** | **P** | **0.04** | **0** | **1** |
| c.650T>G | p.L217X | 2 | MBD2/MBD3 | Nonsense | n.a | n.a | n.a | n.a | 1*PVS, 2*PM, 3*PP, 5*PP | P | 0.04 | 0 | 1 |
| c.764_767dup | **p.V257CfsX13** | **2** | **MBD3** | **Frameshift** | **n.a** | **n.a** | **n.a** | **n.a** | **1*PVS** | **P** | **0.04** | **0** | **1** |
| c.775del | p.L259SfsX2 | 2 | MBD3 | Frameshift | n.a | n.a | n.a | n.a | 1*PVS | P | 0.04 | 0 | 1 |
| c.812dup | **p.C271WfsX5** | **2** | **MBD3** | **Frameshift** | **n.a** | **n.a** | **n.a** | **n.a** | **1*PVS** | **P** | **0.04** | **0** | **1** |
| c.959del | **p.P320LfsX42** | **2** | **MBD3** | **Frameshift** | **n.a** | **n.a** | **n.a** | **n.a** | **1*PVS** | **P** | **0.04** | **0** | **1** |
| c.970A>T | p.K324X | 2 | MBD3 | Nonsense | n.a | n.a | n.a | n.a | 1*PVS, 2*PM, 5*PP | P | 0.04 | 0 | 1 |
| c.976dup | **p.S326FfsX3** | **2** | **MBD3** | **Frameshift** | **n.a** | **n.a** | **n.a** | **n.a** | **1*PVS** | **P** | **0.04** | **0** | **1** |
| c.994G>T | p.E332X | 2 | MBD3/MBD4 | Nonsense | n.a | n.a | n.a | n.a | 1*PVS, 2*PM, 3*PP, 5*PP | P | 0.54 | 2 | 10 |
| c.1117_1127dup | **p.I377VfsX7** | **2** | **MBD4** | **Frameshift** | **n.a** | **n.a** | **n.a** | **n.a** | **1*PVS** | **P** | **0.04** | **0** | **1** |
| c.1121del | **p.V374AfsX6** | **2** | **MBD4** | **Frameshift** | **n.a** | **n.a** | **n.a** | **n.a** | **1*PVS** | **P** | **0.04** | **0** | **1** |
| c.1162C>T | p.Q388X | 2 | MBD4 | Nonsense | n.a | n.a | n.a | n.a | 1*PVS, 2*PM, 3*PP | P | 0.04 | 0 | 1 |
| c.1168A>G | p.I390V | 2 | MBD4 | Missense | 0.492 | Tolerated | 0.001 | Benign | 4*BP, 5*BP | LB | 0.19 | 0 | 5 |
| c.1206_1215dup | **p.S406LfsX1** | **2** | **MBD4** | **Frameshift** | **n.a** | **n.a** | **n.a** | **n.a** | **1*PVS** | **P** | **0.04** | **0** | **1** |
| c.1207_1216dup | **p.S406LfsX1** | **2** | **MBD4** | **Frameshift** | **n.a** | **n.a** | **n.a** | **n.a** | **1*PVS** | **P** | **0.04** | **0** | **1** |
| c.1210_1211dup | **p.N404KfsX4** | **2** | **MBD4** | **Frameshift** | **n.a** | **n.a** | **n.a** | **n.a** | **1*PVS** | **P** | **0.04** | **0** | **1** |
| c.1219_1220del | **p.V407NfsX1** | **2** | **MBD4** | **Frameshift** | **n.a** | **n.a** | **n.a** | **n.a** | **1*PVS** | **P** | **0.04** | **0** | **1** |
| c.1252G>T | **p.E418X** | **2** | **MBD4** | **Nonsense** | **n.a** | **n.a** | **n.a** | **n.a** | **1*PVS, 2*PM, 3*PP** | **P** | **0.04** | **0** | **1** |
| c.1262del | **p.G421DfsX76** | **2** | **MBD4** | **Frameshift** | **n.a** | **n.a** | **n.a** | **n.a** | **1*PVS** | **P** | **0.04** | **0** | **1** |
| c.1297_1307del | **p.T433WfsX68** | **3** | **MBD4/MBD5** | **Frameshift** | **n.a** | **n.a** | **n.a** | **n.a** | **1*PVS** | **P** | **0.04** | **0** | **1** |
| c.1403_1416del | p.A468GfsX32 | 3 | MBD4/MBD5 | Frameshift | n.a | n.a | n.a | n.a | 1*PVS | P | 0.04 | 0 | 1 |
| c.1426G>A | p.A476T | 3 | MBD4/MBD5 | Missense | 0.674 | Tolerated | 0.001 | Benign | 4*BP, 5*PP | LB | 0.04 | 0 | 1 |
| c.1449_1456del | p.R483SfsX20 | 3 | MBD4/MBD5 | Frameshift | n.a | n.a | n.a | n.a | 1*PVS | P | 0.27 | 0 | 7 |
| c.1470C>A | p.C490X | 3 | MBD5 | Nonsense | n.a | n.a | n.a | n.a | 1*PVS, 2*PM, 5*PP | P | 0.23 | 0 | 6 |
| c.1475T>C | p.L492S | 3 | MBD5 | Missense | 0.001 | Damaging | 0.962 | Probably damaging | 3*PS, 2*PM, 3*PP, 5*PP | P | 0.04 | 0 | 1 |
| c.1517_1518del | p.I506RfsX26 | 3 | MBD5 | Frameshift | n.a | n.a | n.a | n.a | 1*PVS | P | 0.04 | 0 | 1 |
| c.1520_1523del | p.E507EfsX15 | 3 | MBD5 | Frameshift | n.a | n.a | n.a | n.a | 1*PVS | P | 0.04 | 0 | 1 |
| c.1529T>C | **p.L510P** | **3** | **MBD5** | **Missense** | **0.005** | **Damaging** | **1.00** | **Probably damaging** | **1*PM, 2*PM, 3*PP** | **LP** | **0.04** | **0** | **1** |
| c.1529T>G | **p.L510R** | **3** | **MBD5** | **Missense** | **0.002** | **Damaging** | **1.00** | **Probably damaging** | **1*PM, 2*PM, 3*PP** | **LP** | **0.04** | **0** | **1** |
| c.1531C>T | p.Q511X | 3 | MBD5 | Nonsense | n.a | n.a | n.a | n.a | 1*PVS, 2*PM, 3*PP, 5*PP | P | 0.23 | 1 | 4 |
| c.1543+1G>T | n.a | 3 | MBD5 | Splicing | n.a | n.a | n.a | n.a | 1*PVS | P | 0.46 | 0 | 12 |
| c.1543+4A>G | n.a | 3 | MBD5 | Splicing | n.a | n.a | n.a | n.a | n.a | US | 0.04 | 0 | 1 |
| c.1571T>C | **p.M524T** | **4** | **MBD5** | **Missense** | **0.012** | **Damaging** | **0.976** | **Probably damaging** | **1*PM, 2*PM, 3*PP** | **LP** | **0.08** | **0** | **2** |
| c.1586A>G | p.E529G | 4 | MBD5 | Missense | 0.001 | Damaging | 1.000 | Probably damaging | 1*PM, 2*PM, 3*PP | LP | 0.04 | 0 | 1 |
| c.1595A>G | p.Y532C | 4 | MBD5 | Missense | 0.000 | Damaging | 1.000 | Probably damaging | 1*PM, 2*PM, 3*PP | LP | 0.04 | 0 | 1 |
| c.1604_1605del | **p.E535GfsX16** | **4** | **MBD5** | **Frameshift** | **n.a** | **n.a** | **n.a** | **n.a** | **1*PVS** | **P** | **0.04** | **0** | **1** |
| c.1648_1654del | p.G550RfsX16 | 4 | MBD5 | Frameshift | n.a | n.a | n.a | n.a | 1*PVS | P | 0.04 | 0 | 1 |
| c.1649del | **p.G550VfsX18** | **4** | **MBD5** | **Frameshift** | **n.a** | **n.a** | **n.a** | **n.a** | **1*PVS** | **P** | **0.04** | **0** | **1** |
| c.1697T>A | **p.I566N** | **4** | **MBD5/MBD6** | **Missense** | **0.002** | **Damaging** | **0.772** | **Possibly damaging** | **1*PM, 2*PM, 3*PP** | **LP** | **0.04** | **0** | **1** |
| c.1707+5G>A | n.a | 4 | MBD5/MBD6 | Splicing | n.a | n.a | n.a | n.a | n.a | LP | 0.08 | 0 | 2 |
| c.1708-5T>G | n.a | 5 | MBD5/MBD6 | Splicing | n.a | n.a | n.a | n.a | n.a | LP | 0.88 | 1 | 21 |
| c.1708-1G>C | n.a | 5 | MBD5/MBD6 | Splicing | n.a | n.a | n.a | n.a | 1*PVS | P | 1.15 | 1 | 28 |
| c.1745_1746del | p.I582RfsX24 | 5 | MBD6 | Frameshift | n.a | n.a | n.a | n.a | 1*PVS | P | 0.04 | 0 | 1 |
| c.1772G>A | p.G591D | 5 | MBD6 | Missense | 0.000 | Damaging | 1.000 | Probably damaging | 1*PM, 2*PM, 3*PP, 5*PP | LP | 0.04 | 0 | 1 |
| c.1774A>T | **p.I592F** | **5** | **MBD6** | **Missense** | **0.001** | **Damaging** | **0.988** | **Probably damaging** | **1*PM, 2*PM, 3*PP** | **LP** | **0.08** | **1** | **0** |
| c.1799_1800del | **p.A600DfsX6** | **5** | **MBD6** | **Frameshift** | **n.a** | **n.a** | **n.a** | **n.a** | **1*PVS** | **P** | **0.04** | **0** | **1** |
| c.1803del | p.S602AfsX46 | 5 | MBD6 | Frameshift | n.a | n.a | n.a | n.a | 1*PVS | P | 0.19 | 0 | 5 |
| c.1817T>G | p.V606G | 5 | MBD6 | Missense | 0.005 | Damaging | 0.979 | Probably damaging | 1*PM, 2*PM, 3*PP | LP | 0.12 | 0 | 3 |
| c.1820dup | p.F608VfsX2 | 5 | MBD6 | Frameshift | n.a | n.a | n.a | n.a | 1*PVS | P | 0.04 | 0 | 1 |
| c.1846C>T | p.R616W | 5 | MBD6 | Missense | 0.001 | Damaging | 1.000 | Probably damaging | 3*PS, 2*PM, 3*PP, 5*PP | P | 0.15 | 0 | 4 |
| c.1847G>A | p.R616Q | 5 | MBD6 | Missense | 0.001 | Damaging | 1.000 | Probably damaging | 1*PM, 2*PM, 3*PP, 5*PP | LP | 0.08 | 0 | 2 |
| c.1900A>G | **p.R634G** | **6** | **TM1** | **Missense** | **0.011** | Damaging | **0.038** | Benign | **1*PM, 2*PM, 3*PP** | **LP** | **0.04** | **0** | **1** |
| c.1901G>A | **p.R634K** | **6** | **TM1** | **Missense** | **0.909** | **Tolerated** | **0.001** | **Benign** | **1*PM, 2*PM** | **LP** | **0.08** | **0** | **2** |
| c.1947-2A>C | **n.a** | **7** | **TM1** | **Splicing** | **n.a** | **n.a** | **n.a** | **n.a** | **1*PVS** | **P** | **0.04** | **0** | **1** |
| c.1949_1951del | **p.W650X** | **7** | **TM1** | **Frameshift** | **n.a** | **n.a** | **n.a** | **n.a** | **1*PVS** | **P** | **0.04** | **0** | **1** |
| c.1988C>G | **p.P663R** | **7** | **TM1** | **Missense** | **0.010** | **Damaging** | **0.947** | **Possibly damaging** | **1*PM, 2*PM, 3*PP** | **LP** | **0.12** | **0** | **3** |
| c.2009_2012dup | p.M671IfsX84 | 7 | TM1/TM2 | Frameshift | n.a | n.a | n.a | n.a | 1*PVS | P | 0.04 | 0 | 1 |
| c.2009A>G | **p.Y670C** | **7** | **TM1/TM2** | **Missense** | **0.001** | **Damaging** | **1.000** | **Probably damaging** | **1*PM, 2*PM, 3*PP** | **US** | **0.04** | **0** | **1** |
| c.2012dup | **p.M671IfsX83** | **7** | **TM1/TM2** | **Frameshift** | **n.a** | **n.a** | **n.a** | **n.a** | **1*PVS** | **P** | **0.04** | **0** | **1** |
| c.2038C>T | p.Q680X | 7 | TM1/TM2 | Nonsense | n.a | n.a | n.a | n.a | 1*PVS, 2*PM, 3*PP, 5*PP | P | 0.04 | 0 | 1 |
| c.2057A>C | **p.H686P** | **7** | **TM1/TM2** | **Missense** | **0.183** | **Tolerated** | **0.323** | **Benign** | **1*PM, 2*PM, 3*PP** | **LP** | **0.04** | **0** | **1** |
| c.2075T>C | p.L692P | 7 | TM2 | Missense | 0.000 | Damaging | 1.000 | Probably damaging | 1*PM, 2*PM, 3*PP | LP | 0.08 | 0 | 2 |
| c.2078C>G | p.S693C | 7 | TM2 | Missense | 0.000 | Damaging | 1.000 | Probably damaging | 1*PM, 2*PM, 3*PP | LP | 0.08 | 0 | 2 |
| c.2111C>T | **p.T704I** | **7** | **TM2** | **Missense** | **0.075** | **Tolerated** | **0.988** | **Probably damaging** | **1*PM, 2*PM, 3*PP** | **LP** | **0.04** | **0** | **1** |
| c.2120A>G | p.Q707R | 7 | TM2 | Missense | 0.001 | Damaging | 1.000 | Probably damaging | 1*PM, 2*PM, 3*PP | LP | 0.12 | 0 | 3 |
| c.2122-1G>C | **n.a** | **8** | **TM2** | **Splicing** | **n.a** | **n.a** | **n.a** | **n.a** | **1*PVS** | **P** | **0.08** | **0** | **2** |
| c.2122-1G>T | **n.a** | **8** | **TM2** | **Splicing** | **n.a** | **n.a** | **n.a** | **n.a** | **1*PVS** | **P** | **0.15** | **0** | **4** |
| c.2128G>A | p.G710S | 8 | TM2/TM3 | Missense | 0.000 | Damaging | 1.000 | Probably damaging | 3*PS, 2*PM, 3*PP, 5*PP | P | 0.15 | 0 | 4 |
| c.2139C>G | **p.Y713X** | **8** | **TM2/TM3** | **Nonsense** | **n.a** | **n.a** | **n.a** | **n.a** | **1*PVS, 2*PM, 3*PP** | **LP** | **0.04** | **0** | **1** |
| c.2141T>A | **p.F714Y** | **8** | **TM2/TM3** | **Missense** | **0.000** | **Damaging** | **1.000** | **Probably damaging** | **1*PM, 2*PM, 3*PP** | **LP** | **0.04** | **0** | **1** |
| c.2142C>G | **p.F714L** | **8** | **TM2/TM3** | **Missense** | **0.000** | **Damaging** | **1.000** | **Probably damaging** | **1*PM, 2*PM** | **LP** | **0.04** | **0** | **1** |
| c.2145C>A | p.Y715X | 8 | TM2/TM3 | Nonsense | n.a | n.a | n.a | n.a | 1*PVS, 2*PM, 5*PP | P | 0.35 | 1 | 7 |
| c.2165dup | p.R723EfsX31 | 8 | TM2/TM3 | Frameshift | n.a | n.a | n.a | n.a | 1*PVS | P | 0.04 | 0 | 1 |
| c.2185A>G | p.M729V | 8 | TM3 | Missense | 0.000 | Damaging | 1.000 | Probably damaging | 1*PM, 2*PM, 3*PP | LP | 0.04 | 0 | 1 |
| c.2187G>A | **p.M729I** | **8** | **TM3** | **Missense** | **0.000** | **Damaging** | **1.000** | **Probably damaging** | **1*PM, 2*PM, 3*PP** | **LP** | **0.08** | **0** | **2** |
| c.2195T>C | p.L732P | 8 | TM3 | Missense | 0.000 | Damaging | 1.000 | Probably damaging | 1*PM, 2*PM, 3*PP | LP | 0.08 | 0 | 2 |
| c.2227del | p.Y743IfsX18 | 8 | TM3 | Frameshift | n.a | n.a | n.a | n.a | 1*PVS | P | 0.04 | 0 | 1 |
| c.2230T>A | **p.S744T** | **8** | **TM3** | **Missense** | **0.000** | **Damaging** | **1.000** | **Probably damaging** | **1*PM, 2*PM, 3*PP** | **LP** | **0.04** | **0** | **1** |
| c.2230T>C | p.S744P | 8 | TM3 | Missense | 0.000 | Damaging | 1.000 | Probably damaging | 1*PM, 2*PM, 3*PP, 5*PP | LP | 0.04 | 0 | 1 |
| c.2231C>T | **p.S744F** | **8** | **TM3** | **Missense** | **0.000** | **Damaging** | **1.000** | **Probably damaging** | **1*PM, 2*PM, 3*PP** | **LP** | **0.04** | **0** | **1** |
| c.2252C>A | **p.A751D** | **8** | **TM3/TM4** | **Missense** | **0.001** | **Damaging** | **1.000** | **Probably damaging** | **1*PM, 2*PM, 3*PP** | **LP** | **0.15** | **0** | **4** |
| c.2260G>C | **p.E754Q** | **8** | **TM3/TM4** | **Missense** | **0.157** | **Tolerated** | **0.974** | **Probably damaging** | **1*PM, 2*PM, 3*PP** | **LP** | **0.04** | **0** | **1** |
| c.2261A>G | p.E754G | 8 | TM3/TM4 | Missense | 0.076 | Tolerated | 0.998 | Probably damaging | 1*PM, 2*PM | LP | 0.04 | 0 | 1 |
| c.2267C>G | p.A756G | 8 | TM3/TM4 | Missense | 0.051 | Tolerated | 1.000 | Probably damaging | 1*PM, 2*PM, 3*PP | LP | 0.08 | 0 | 2 |
| c.2276G>A | **p.S759N** | **8** | **TM3/TM4** | **Missense** | **0.378** | **Tolerated** | **0.709** | **Possibly damaging** | **2*PM, 3*PP** | **LP** | **0.04** | **0** | **1** |
| c.2278C>T | **p.P760S** | **8** | **TM3/TM4** | **Missense** | **0.002** | **Damaging** | **1.000** | **Probablydamaging** | **2*PM, 3*PP** | **LP** | **0.04** | **0** | **1** |
| c.2286dup | **p.F763IfsX31** | **8** | **TM4** | **Frameshift** | **n.a** | **n.a** | **n.a** | **n.a** | **1*PVS** | **P** | **0.04** | **0** | **1** |
| c.2293G>A | p.D765N | 8 | TM4 | Missense | 0.001 | Damaging | 1.000 | Probably damaging | 3*PS, 2*PM, 3*PP, 5*PP | P | 0.19 | 0 | 5 |
| c.2294A>G | p.D765G | 8 | TM4 | Missense | 0.001 | Damaging | 1.000 | Probably damaging | 1*PM, 2*PM, 3*PP, 5*PP | LP | 0.81 | 0 | 21 |
| c.2297C>T | p.T766M | 8 | TM4 | Missense | 0.001 | Damaging | 1.000 | Probably damaging | 1*PM, 2*PM, 5*PM, 3*PP | LP | 0.08 | 0 | 2 |
| c.2304dup | p.M769HfsX26 | 8 | TM4 | Frameshift | n.a | n.a | n.a | n.a | 1*PVS | P | 1.69 | 2 | 40 |
| c.2305A>G | p.M769V | 8 | TM4 | Missense | 0.000 | Damaging | 1.000 | Probably damaging | 3*PS, 2*PM, 3*PP, 5*PP | P | 0.04 | 0 | 1 |
| c.2308C>T | p.L770F | 8 | TM4 | Missense | 0.000 | Damaging | 1.000 | Probably damaging | 1*PM, 2*PM, 3*PP | LP | 0.08 | 0 | 2 |
| c.2327T>C | p.L776P | 8 | TM4 | Missense | 0.000 | Damaging | 1.000 | Probably damaging | 1*PM, 2*PM, 3*PP | LP | 0.08 | 0 | 2 |
| c.2332C>T | p.R778W | 8 | TM4 | Missense | 0.000 | Damaging | 1.000 | Probably damaging | 3*PS, 2*PM, 3*PP, 5*PP | P | 0.38 | 0 | 10 |
| c.2333G>A | p.R778Q | 8 | TM4 | Missense | 0.000 | Damaging | 1.000 | Probably damaging | 3*PS, 2*PM, 3*PP, 5*PP | P | 0.42 | 0 | 11 |
| c.2333G>T | p.R778L | 8 | TM4 | Missense | 0.000 | Damaging | 1.000 | Probably damaging | 3*PS, 2*PM, 3*PP, 5*PP | P | 28.96 | 125 | 504 |
| c.2336G>A | p.W779X | 8 | TM4 | Nonsense | n.a | n.a | n.a | n.a | 1*PVS, 2*PM, 3*PP, 5*PP | P | 0.15 | 0 | 4 |
| c.2337G>A | p.W779X | 8 | TM4 | Nonsense | n.a | n.a | n.a | n.a | 1*PVS, 2*PM, 3*PP | P | 0.04 | 0 | 1 |
| c.2356-2A>G | n.a | 9 | TM4/A-domain | Splicing | n.a | n.a | n.a | n.a | 1*PVS | P | 0.15 | 0 | 4 |
| c.2375T>C | **p.L792P** | **9** | **TM4/A-domain** | **Missense** | **0.000** | **Damaging** | **0.997** | **Probably damaging** | **1*PM, 2*PM, 3*PP** | **LP** | **0.04** | **0** | **1** |
| c.2383C>T | p.L795F | 9 | TM4/A-domain | Missense | 0.000 | Damaging | 1.000 | Probably damaging | 1*PM, 2*PM, 3*PP, 5*PP | LP | 0.04 | 0 | 1 |
| c.2395C>G | **p.Q799E** | **9** | **TM4/A-domain** | **Missense** | **0.003** | **Damaging** | **1.000** | **Probably damaging** | **1*PM, 2*PM, 3*PP** | **LP** | **0.04** | **0** | **1** |
| c.2419A>C | **p.T807P** | **9** | **TM4/A-domain** | **Missense** | **0.005** | **Damaging** | **0.996** | **Probably damaging** | **1*PM, 2*PM, 3*PP** | **LP** | **0.04** | **0** | **1** |
| c.2438T>G | **p.L813X** | **9** | **TM4/A-domain** | **Nonsense** | **n.a** | **n.a** | **n.a** | **n.a** | **1*PVS, 2*PM, 3*PP** | **P** | **0.04** | **0** | **1** |
| c.2447+5G>T | n.a | 9 | TM4/A-domain | Splicing | n.a | n.a | n.a | n.a | n.a | LP | 0.19 | 0 | 5 |
| c.2448-5T>G | **n.a** | **10** | **A-domain** | **Splicing** | **n.a** | **n.a** | **n.a** | **n.a** | **n.a** | **US** | **0.04** | **0** | **1** |
| c.2455C>T | p.Q819X | 10 | A-domain | Nonsense | n.a | n.a | n.a | n.a | 1*PVS**,** 2*PM, 3*PP | P | 0.08 | 0 | 2 |
| c.2471T>G | **p.L824R** | **10** | **A-domain** | **Missense** | **0.000** | **Damaging** | **0.993** | **Probably damaging** | **1*PM, 2*PM, 3*PP** | **LP** | **0.04** | **0** | **1** |
| c.2513A>C | **p.K838T** | **10** | **A-domain** | **Missense** | **0.001** | **Damaging** | **0.913** | **Possibly damaging** | **1*PM, 2*PM, 3*PP** | **LP** | **0.08** | **0** | **2** |
| c.2539G>A | **p.E847K** | **10** | **A-domain** | **Missense** | **0.053** | **Tolerated** | **0.995** | **Probably damaging** | **1*PM, 2*PM, 3*PP** | **LP** | **0.04** | **0** | **1** |
| c.2549C>T | p.T850I | 10 | A-domain | Missense | 0.001 | Damaging | 0.037 | Benign | 1*PM, 2*PM, 3*PP, 5*PP | LP | 0.08 | 0 | 2 |
| c.2561A>T | p.E854V | 10 | A-domain | Missense | 0.000 | Damaging | 0.992 | Probably damaging | 1*PM, 2*PM, 3*PP | LP | 0.08 | 1 | 0 |
| c.2590_2593dup | p.T865SfsX3 | 11 | A-domain | Frameshift | n.a | n.a | n.a | n.a | 1*PVS | P | 0.12 | 0 | 3 |
| c.2604del | p.G869EfsX3 | 11 | A-domain/TM5 | Frameshift | n.a | n.a | n.a | n.a | 1*PVS | P | 0.15 | 1 | 2 |
| c.2605G>A | p.G869R | 11 | A-domain/TM5 | Missense | 0.001 | Damaging | 1.000 | Probably damaging | 1*PM, 2*PM, 3*PP | LP | 0.04 | 0 | 1 |
| c.2620G>C | p.A874P | 11 | A-domain/TM5 | Missense | 0.006 | Damaging | 0.999 | Probably damaging | 1*PM, 2*PM, 3*PP, 5*PP | LP | 0.73 | 2 | 15 |
| c.2621C>T | p.A874V | 11 | A-domain/TM5 | Missense | 0.004 | Damaging | 0.999 | Probably damaging | 3*PS, 1*PM, 2*PM, 3*PP | P | 5.99 | 6 | 144 |
| c.2642G>A | **p.G881D** | **11** | **A-domain/TM5** | **Missense** | **0.001** | **Damaging** | **0.999** | **Probably damaging** | **1*PM, 2*PM, 3*PP** | **LP** | **0.04** | **0** | **1** |
| c.2659del | p.A887LfsX14 | 11 | A-domain/TM5 | Frameshift | n.a | n.a | n.a | n.a | 1*PVS | P | 0.19 | 0 | 5 |
| c.2662A>C | p.T888P | 11 | A-domain/TM5 | Missense | 0.001 | Damaging | 1.000 | Probably damaging | 1*PM, 2*PM, 3*PP, 5*PP | LP | 0.84 | 1 | 20 |
| c.2668G>A | p.V890M | 11 | A-domain/TM5 | Missense | 0.003 | Damaging | 1.000 | Probably damaging | 1*PM, 2*PM, 3*PP | LP | 0.46 | 1 | 10 |
| c.2696T>C | **p.I899T** | **11** | **A-domain/TM5** | **Missense** | **0.000** | **Damaging** | **1.000** | **Probably damaging** | **1*PM, 2*PM, 3*PP** | **LP** | **0.04** | **0** | **1** |
| c.2697_2723del | p.899_907del | 11 | A-domain/TM5 | Inframe del | n.a | n.a | n.a | n.a | 2*PM, 4*PM, 5*PP | LP | 0.12 | 0 | 3 |
| c.2705T>C | **p.L902P** | **11** | **A-domain/TM5** | **Missense** | **0.000** | **Damaging** | **1.000** | **Probably damaging** | **1*PM, 2*PM, 3*PP** | **LP** | **0.04** | **0** | **1** |
| c.2731-1G>A | **n.a** | **12** | **A-domain/TM5** | **Splicing** | **n.a** | **n.a** | **n.a** | **n.a** | **1*PVS** | **P** | **0.04** | **0** | **1** |
| c.2731G>A | **p.A911T** | **12** | **A-domain/TM5** | **Missense** | **0.000** | **Damaging** | **1.000** | **Probably damaging** | **1*PM, 2*PM, 3*PP** | **LP** | **0.04** | **0** | **1** |
| c.2752G>A | p.D918N | 12 | TM5 | Missense | 0.000 | Damaging | 1.000 | Probably damaging | 1*PM, 2*PM, 3*PP | LP | 0.08 | 0 | 2 |
| c.2755C>G | p.R919G | 12 | TM5 | Missense | 0.002 | Damaging | 0.832 | Possibly damaging | 3*PS, 2*PM, 3*PP, 5*PP | P | 2.46 | 0 | 64 |
| c.2790_2792del | p.I930del | 12 | TM5 | Inframe del | n.a | n.a | n.a | n.a | 2*PM, 4*PM, 5*PP | LP | 0.50 | 0 | 13 |
| c.2795C>A | p.S932X | 12 | TM5 | Nonsense | n.a | n.a | n.a | n.a | 1*PVS, 2*PM, 3*PP, 5*PP | P | 0.04 | 0 | 1 |
| c.2804C>T | p.T935M | 12 | TM5 | Missense | 0.000 | Damaging | 1.000 | Probably damaging | 1*PM, 2*PM, 3*PP, 5*PP | LP | 0.92 | 0 | 24 |
| c.2806_2808del | p.L936del | 12 | TM5 | Inframe del | n.a | n.a | n.a | n.a | 2*PM, 4*PM, 5*PP | LP | 0.04 | 0 | 1 |
| c.2810del | p.V937GfsX5 | 12 | TM5 | Frameshift | n.a | n.a | n.a | n.a | 1*PVS | P | 0.15 | 0 | 4 |
| c.2827G>A | p.G943S | 12 | TM5/TM6 | Missense | 0.000 | Damaging | 1.000 | Probably damaging | 1*PM, 2*PM, 3*PP, 5*PP | LP | 0.54 | 1 | 12 |
| c.2828G>A | p.G943D | 12 | TM5/TM6 | Missense | 0.000 | Damaging | 1.000 | Probably damaging | 1*PM, 2*PM, 3*PP, 5*PP | LP | 0.38 | 0 | 10 |
| c.2887C>T | p.Q963X | 13 | TM5/TM6 | Nonsense | n.a | n.a | n.a | n.a | 1*PVS, 2*PM, 3*PP | P | 0.04 | 0 | 1 |
| c.2891_2894del | **p.T964RfsX1** | **13** | **TM5/TM6** | **Frameshift** | **n.a** | **n.a** | **n.a** | **n.a** | **1*PVS** | **P** | **0.04** | **0** | **1** |
| c.2894_2895del | **p.E965GfsX61** | **13** | **TM5/TM6** | **Frameshift** | **n.a** | **n.a** | **n.a** | **n.a** | **1*PVS** | **P** | **0.08** | **0** | **2** |
| c.2903T>A | **p.I968N** | **13** | **TM6** | **Missense** | **0.001** | **Damaging** | **0.977** | **Probably damaging** | **1*PM, 2*PM, 3*PP** | **LP** | **0.04** | **0** | **1** |
| c.2905C>T | p.R969W | 13 | TM6 | Missense | 0.016 | Damaging | 1.000 | Probably damaging | 1*PM, 2*PM, 3*PP, 5*PP | LP | 0.04 | 0 | 1 |
| c.2906G>A | p.R969Q | 13 | TM6 | Missense | 0.382 | Tolerated | 1.000 | Probably damaging | 3*PS, 2*PM, 3*PP, 5*PP | P | 0.15 | 0 | 4 |
| c.2924C>A | p.S975Y | 13 | TM6 | Missense | 0.001 | Damaging | 1.000 | Probably damaging | 1*PM, 2*PM, 3*PP, 5*PP | LP | 1.46 | 3 | 32 |
| c.2930C>T | p.T977M | 13 | TM6 | Missense | 0.001 | Damaging | 1.000 | Probably damaging | 1*PM, 2*PM, 3*PP, 5*PP | LP | 0.23 | 0 | 6 |
| c.2936T>C | **p.L979P** | **13** | **TM6** | **Missense** | **0.000** | **Damaging** | **1.000** | **Probably damaging** | **1*PM, 2*PM, 3*PP** | **LP** | **0.04** | **0** | **1** |
| c.2938T>C | p.C980R | 13 | TM6 | Missense | 0.003 | Damaging | 1.000 | Probably damaging | 1*PM, 2*PM, 3*PP | LP | 0.08 | 0 | 2 |
| c.2939G>A | p.C980Y | 13 | TM6 | Missense | 0.001 | Damaging | 0.997 | Probably damaging | 1*PM, 2*PM, 3*PP, 5*PP | LP | 0.12 | 0 | 3 |
| c.2957C>T | p.S986F | 13 | TM6 | Missense | 0.001 | Damaging | 1.000 | Probably damaging | 1*PM, 2*PM, 3*PP | LP | 0.04 | 0 | 1 |
| c.2963G>T | p.G988V | 13 | TM6 | Missense | 0.001 | Damaging | 1.000 | Probably damaging | 1*PM, 2*PM, 5*PM, 3*PP | LP | 0.19 | 0 | 5 |
| c.2975C>T | p.P992L | 13 | TM6 | Missense | 0.001 | Damaging | 1.000 | Probably damaging | 3*PS, 2*PM, 3*PP, 5*PP | P | 13.82 | 39 | 282 |
| c.2998G>A | p.G1000R | 13 | TM6 | Missense | 0.000 | Damaging | 1.000 | Probably damaging | 1*PM, 2*PM, 3*PP | LP | 0.08 | 0 | 2 |
| c.3004G>A | **p.A1002T** | **13** | **TM6** | **Missense** | **0.002** | **Damaging** | **0.996** | **Probably damaging** | **1*PM, 2*PM, 3*PP** | **LP** | **0.04** | **0** | **1** |
| c.3007G>A | p.A1003T | 13 | TM6/P-domain | Missense | 0.001 | Damaging | 1.000 | Probably damaging | 1*PM, 2*PM, 3*PP, 5*PP | LP | 0.15 | 0 | 4 |
| c.3008C>T | p.A1003V | 13 | TM6/P-domain | Missense | 0.001 | Damaging | 1.000 | Probably damaging | 1*PM, 2*PM, 3*PP, 5*PP | LP | 0.08 | 0 | 2 |
| c.3028A>G | p.K1010E | 13 | TM6/P-domain | Missense | 0.000 | Damaging | 1.000 | Probably damaging | 1*PM, 2*PM, 3*PP | LP | 0.19 | 0 | 5 |
| c.3029A>C | p.K1010T | 13 | TM6/P-domain | Missense | 0.000 | Damaging | 0.999 | Probably damaging | 1*PM, 2*PM, 5*PM, 3*PP | LP | 0.15 | 0 | 4 |
| c.3044T>C | p.L1015P | 13 | TM6/P-domain | Missense | 0.000 | Damaging | 1.000 | Probably damaging | 1*PM, 2*PM, 3*PP | LP | 0.15 | 0 | 4 |
| c.3044T>G | **p.L1015R** | **13** | **TM6/P-domain** | **Missense** | **0.000** | **Damaging** | **1.000** | **Probably damaging** | **1*PM, 2*PM, 3*PP** | **LP** | **0.04** | **0** | **1** |
| c.3052G>A | **p.A1018T** | **13** | **TM6/P-domain** | **Missense** | **0.031** | **Damaging** | **0.998** | **Probably damaging** | **1*PM, 2*PM, 3*PP** | **LP** | **0.08** | **0** | **2** |
| c.3053C>T | p.A1018V | 13 | TM6/P-domain | Missense | 0.060 | Tolerated | 0.999 | Probably damaging | 1*PM, 2*PM, 3*PP, 5*PP | LP | 0.12 | 0 | 3 |
| c.3056A>C | p.H1019P | 13 | P-domain | Missense | 0.002 | Damaging | 1.000 | Probably damaging | 1*PM, 2*PM, 3*PP | LP | 0.04 | 0 | 1 |
| c.3056A>T | **p.H1019L** | **13** | P-domain | **Missense** | **0.002** | **Damaging** | **0.999** | **Probably damaging** | **1*PM, 2*PM, 3*PP** | **LP** | **0.04** | **0** | **1** |
| c.3060+5G>T | n.a | 13 | P-domain | Splicing | n.a | n.a | n.a | n.a | n.a | LP | 0.04 | 0 | 1 |
| c.3061-3C>A | n.a | 14 | P-domain | Splicing | n.a | n.a | n.a | n.a | n.a | LP | 0.08 | 1 | 0 |
| c.3071_3072del | **p.V1024DfsX2** | **14** | **P-domain** | **Frameshift** | **n.a** | **n.a** | **n.a** | **n.a** | **1*PVS** | **P** | **0.04** | **0** | **1** |
| c.3079G>C | **p.D1027H** | **14** | **P-domain** | **Missense** | **0.001** | **Damaging** | **1.000** | **Probably damaging** | **1*PM, 2*PM, 3*PP** | **LP** | **0.08** | **0** | **2** |
| c.3089G>A | p.G1030D | 14 | P-domain | Missense | 0.000 | Damaging | 1.000 | Probably damaging | 1*PM, 2*PM, 3*PP | LP | 0.27 | 0 | 7 |
| c.3104G>T | p.G1035V | 14 | N-domain | Missense | 0.000 | Damaging | 1.000 | Probably damaging | 1*PM, 2*PM, 3*PP, 5*PP | LP | 0.08 | 0 | 2 |
| c.3121C>T | p.R1041W | 14 | N-domain | Missense | 0.001 | Damaging | 1.000 | Probably damaging | 1*PM, 2*PM, 5*PP | LP | 0.08 | 0 | 2 |
| c.3140A>T | p.D1047V | 14 | N-domain | Missense | 0.019 | Damaging | 0.992 | Probably damaging | 1*PM, 2*PM, 3*PP | LP | 0.19 | 1 | 3 |
| c.3155C>T | p.P1052L | 14 | N-domain | Missense | 0.022 | Damaging | 0.963 | Probably damaging | 3*PS, 1*PM, 2*PM, 3*PP | P | 0.04 | 0 | 1 |
| c.3173C>A | **p.A1058D** | **14** | **N-domain** | **Missense** | **0.002** | **Damaging** | **1.000** | **Probably damaging** | **1*PM, 2*PM, 3*PP** | **LP** | **0.04** | **0** | **1** |
| c.3181G>T | **p.G1061W** | **14** | **N-domain** | **Missense** | **0.006** | **Damaging** | **0.978** | **Probably damaging** | **1*PM, 2*PM, 3*PP** | **LP** | **0.04** | **0** | **1** |
| c.3212T>C | **p.L1071S** | **14** | **N-domain** | **Missense** | **0.001** | **Damaging** | **1.000** | **Probably damaging** | **1*PM, 2*PM, 3*PP** | **LP** | **0.04** | **0** | **1** |
| c.3220G>A | **p.A1074T** | **14** | **N-domain** | **Missense** | **0.001** | **Damaging** | **1.000** | **Probably damaging** | **1*PM, 2*PM, 3*PP** | **LP** | **0.04** | **0** | **1** |
| c.3229_3236dup | **p.C1079X** | **14** | **N-domain** | **Frameshift** | **n.a** | **n.a** | **n.a** | **n.a** | **1*PVS** | **P** | **0.04** | **0** | **1** |
| c.3236G>A | **p.C1079Y** | **14** | **N-domain** | **Missense** | **0.000** | **Damaging** | **1.000** | **Probably damaging** | **1*PM, 2*PM, 5*PM, 3*PP** | **LP** | **0.04** | **0** | **1** |
| c.3243G>A | p.E1081E | 14 | N-domain | Splicing | n.a | n.a | n.a | n.a | 1*PVS, 2*PM, 3*PP | P | 0.08 | 0 | 2 |
| c.3243+5G>A | n.a | 14 | N-domain | Splicing | n.a | n.a | n.a | n.a | n.a | LP | 0.04 | 0 | 1 |
| c.3244-2A>G | n.a | 15 | N-domain | Splicing | n.a | n.a | n.a | n.a | 1*PVS | P | 0.15 | 0 | 4 |
| c.3263T>C | p.L1088S | 15 | N-domain | Missense | 0.071 | Tolerated | 1.000 | Probably damaging | 1*PM, 2*PM, 3*PP | LP | 0.27 | 0 | 7 |
| c.3271T>C | p.C1091R | 15 | N-domain | Missense | 0.001 | Damaging | 0.979 | Probably damaging | 1*PM, 2*PM, 3*PP | LP | 0.04 | 0 | 1 |
| c.3274A>C | p.T1092P | 15 | N-domain | Missense | 0.041 | Damaging | 0.860 | Possibly damaging | 1*PM, 2*PM, 3*PP | LP | 0.04 | 0 | 1 |
| c.3287C>A | **p.A1096E** | **15** | **N-domain** | **Missense** | **0.003** | **Damaging** | **0.998** | **Probably damaging** | **1*PM, 2*PM, 3*PP** | **LP** | **0.04** | **0** | **1** |
| c.3310T>C | p.C1104R | 15 | N-domain | Missense | 0.000 | Damaging | 1.000 | Probably damaging | 1*PM, 2*PM, 3*PP | LP | 0.08 | 0 | 2 |
| c.3316G>A | p.V1106I | 15 | N-domain | Missense | 0.156 | Tolerated | 0.998 | Probably damaging | 1*PM, 2*PM, 3*PP | LP | 1.57 | 0 | 41 |
| c.3326T>C | **p.V1109A** | **15** | **N-domain** | **Missense** | **0.045** | **Damaging** | **0.978** | **Probably damaging** | **1*PM, 2*PM, 3*PP** | **LP** | **0.04** | **0** | **1** |
| c.3348dup | **p.E1117X** | **15** | **N-domain** | **Frameshift** | **n.a** | **n.a** | **n.a** | **n.a** | **1*PVS** | **P** | **0.04** | **0** | **1** |
| c.3368del | **p.P1123RfsX4** | **15** | **N-domain** | **Frameshift** | **n.a** | **n.a** | **n.a** | **n.a** | **1*PVS** | **P** | **0.04** | **0** | **1** |
| c.3377_3378del | p.H1126PfsX3 | 15 | N-domain | Frameshift | n.a | n.a | n.a | n.a | 1*PVS | P | 0.12 | 0 | 3 |
| c.3384del | **p.N1128KfsX19** | **15** | **N-domain** | **Frameshift** | **n.a** | **n.a** | **n.a** | **n.a** | **1*PVS** | **P** | **0.04** | **0** | **1** |
| c.3424C＞T | p.Q1142X | 16 | N-domain | Nonsense | n.a | n.a | n.a | n.a | 1*PVS, 2*PM, 3*PP | P | 0.04 | 0 | 1 |
| c.3443T>A | **p.I1148N** | **16** | **N-domain** | **Missense** | **0.000** | **Damaging** | **1.000** | **Probably damaging** | **1*PM, 2*PM, 5*PM, 3*PP** | **LP** | **0.04** | **0** | **1** |
| c.3443T>C | p.I1148T | 16 | N-domain | Missense | 0.001 | Damaging | 1.000 | Probably damaging | 1*PM, 2*PM, 3*PP | LP | 1.73 | 3 | 39 |
| c.3446G>A | p.G1149E | 16 | N-domain | Missense | 0.000 | Damaging | 1.000 | Probably damaging | 1*PM, 2*PM, 5*PM, 3*PP | LP | 0.27 | 0 | 7 |
| c.3449A>T | **p.N1150I** | **16** | **N-domain** | **Missense** | **0.000** | **Damaging** | **1.000** | **Probably damaging** | **1*PM, 2*PM, 3*PP** | **LP** | **0.04** | **0** | **1** |
| c.3450C>A | **p.N1150K** | **16** | **N-domain** | **Missense** | **0.001** | **Damaging** | **0.999** | **Probably damaging** | **1*PM, 2*PM, 3*PP** | **LP** | **0.04** | **0** | **1** |
| c.3451C>T | p.R1151C | 16 | N-domain | Missense | 0.000 | Damaging | 1.000 | Probably damaging | 1*PM, 2*PM, 3*PP, 5*PP | LP | 0.08 | 0 | 2 |
| c.3452G>A | p.R1151H | 16 | N-domain | Missense | 0.001 | Damaging | 1.000 | Probably damaging | 1*PM, 2*PM, 3*PP | LP | 0.04 | 0 | 1 |
| c.3459G>T | p.W1153C | 16 | N-domain | Missense | 0.001 | Damaging | 1.000 | Probably damaging | 1*PM, 2*PM, 3*PP | LP | 0.35 | 0 | 9 |
| c.3461T>G | **p.L1154R** | **16** | **N-domain** | **Missense** | **0.000** | **Damaging** | **0.987** | **Probably damaging** | **1*PM, 2*PM, 3*PP** | **LP** | **0.04** | **0** | **1** |
| c.3505A>G | p.M1169V | 16 | N-domain | Missense | 0.002 | Damaging | 0.965 | Probably damaging | 1*PM, 2*PM, 3*PP, 5*PP | LP | 0.08 | 0 | 2 |
| c.3517G>A | p.E1173K | 16 | N-domain | Missense | 0.002 | Damaging | 0.994 | Probably damaging | 1*PM, 2*PM, 3*PP, 5*PP | LP | 0.88 | 0 | 23 |
| c.3532A>G | p.T1178A | 16 | N-domain | Missense | 0.003 | Damaging | 0.997 | Probably damaging | 1*PM, 2*PM, 3*PP, 5*PP | LP | 0.27 | 0 | 7 |
| c.3551T>C | p.I1184T | 16 | N-domain | Missense | 0.001 | Damaging | 0.999 | Probably damaging | 1*PM, 2*PM, 3*PP | LP | 0.04 | 0 | 1 |
| c.3556G>A | p.G1186S | 16 | N-domain | Missense | 0.045 | Damaging | 0.999 | Probably damaging | 1*PM, 2*PM, 3*PP, 5*PP | LP | 0.08 | 0 | 2 |
| c.3556+1G>A | n.a | 16 | N-domain | Splicing | n.a | n.a | n.a | n.a | 1*PVS | P | 0.04 | 0 | 1 |
| c.3557-2A>G | n.a | 17 | N-domain | Splicing | n.a | n.a | n.a | n.a | 1*PVS | P | 0.04 | 0 | 1 |
| c.3562C>T | **p.L1188F** | **17** | **N-domain** | **Missense** | **0.002** | **Damaging** | 1.000 | **Probably damaging** | **1*PM, 2*PM, 3*PP** | **LP** | **0.15** | **0** | **4** |
| c.3587A>G | p.D1196G | 17 | P-domain | Missense | 0.000 | Damaging | 1.000 | Probably damaging | 1*PM, 2*PM, 3*PP | LP | 0.12 | 0 | 3 |
| c.3646G>A | p.V1216M | 17 | P-domain | Missense | 0.001 | Damaging | 1.000 | Probably damaging | 1*PM, 2*PM, 3*PP, 5*PP | LP | 1.92 | 3 | 44 |
| c.3659C>G | **p.T1220R** | **17** | **P-domain** | **Missense** | **0.000** | **Damaging** | **1.000** | **Probably damaging** | **1*PM, 2*PM, 5*PM, 3*PP** | **LP** | **0.04** | **0** | **1** |
| c.3662_3664del | p.G1221del | 17 | P-domain | Inframe del | n.a | n.a | n.a | n.a | 4*PM | LP | 0.12 | 0 | 3 |
| c.3680C>A | **p.A1227D** | **17** | **P-domain** | **Missense** | **0.001** | **Damaging** | **1.000** | **Probably damaging** | **1*PM, 2*PM, 3*PP** | **LP** | **0.04** | **0** | **1** |
| c.3686C>G | **p.A1229G** | **17** | **P-domain** | **Missense** | **0.006** | **Damaging** | **0.997** | **Probably damaging** | **1*PM, 2*PM, 3*PP** | **LP** | **0.04** | **0** | **1** |
| c.3699+2T>C | **n.a** | **17** | **P-domain** | **Splicing** | **n.a** | **n.a** | **n.a** | **n.a** | **1*PVS** | **P** | **0.04** | **0** | **1** |
| c.3700-3T>G | **n.a** | **18** | **P-domain** | **Splicing** | **n.a** | **n.a** | **n.a** | **n.a** | **n.a** | **US** | **0.04** | **0** | **1** |
| c.3700-1G>A | n.a | 18 | P-domain | Splicing | n.a | n.a | n.a | n.a | 1*PVS | P | 0.08 | 0 | 2 |
| c.3700del | p.V1234LfsX96 | 18 | P-domain | Frameshift | n.a | n.a | n.a | n.a | 1*PVS | P | 0.61 | 0 | 16 |
| c.3707T>C | p.I1236T | 18 | P-domain | Missense | 0.000 | Damaging | 1.000 | Probably damaging | 1*PM, 2*PM, 3*PP | LP | 0.04 | 0 | 1 |
| c.3715G>T | p.V1239F | 18 | P-domain | Missense | 0.001 | Damaging | 1.000 | Probably damaging | 1*PM, 2*PM, 3*PP | LP | 0.04 | 0 | 1 |
| c.3724G>A | **p.E1242K** | **18** | **P-domain** | **Missense** | **0.002** | **Damaging** | **0.999** | **Probably damaging** | **1*PM, 2*PM, 3*PP** | **LP** | **0.04** | **0** | **1** |
| c.3733C>G | p.P1245A | 18 | P-domain | Missense | 0.000 | Damaging | 1.000 | Probably damaging | 1*PM, 2*PM, 3*PP | LP | 0.04 | 0 | 1 |
| c.3744G>C | p.K1248N | 18 | P-domain | Missense | 0.000 | Damaging | 1.000 | Probably damaging | 1*PM, 2*PM, 3*PP | LP | 0.04 | 0 | 1 |
| c.3766_3767dup | p.Q1256HfsX75 | 18 | P-domain | Frameshift | n.a | n.a | n.a | n.a | 1*PVS | P | 0.15 | 0 | 4 |
| c.3794_3797dup | **p.D1267GfsX5** | **18** | **P-domain** | **Frameshift** | **n.a** | **n.a** | **n.a** | **n.a** | **1*PVS** | **P** | **0.04** | **0** | **1** |
| c.3796G>C | p.G1266R | 18 | P-domain | Missense | 0.000 | Damaging | 1.000 | Probably damaging | 1*PS, 3*PS, 2*PM, 3*PP | P | 0.04 | 0 | 1 |
| c.3799G>A | p.D1267N | 18 | P-domain | Missense | 0.000 | Damaging | 0.997 | Probably damaging | 1*PM, 2*PM, 3*PP | LP | 0.04 | 0 | 1 |
| c.3800A>G | p.D1267G | 18 | P-domain | Missense | 0.000 | Damaging | 1.000 | Probably damaging | 1*PM, 2*PM,5*PM, 3*PP | LP | 0.04 | 0 | 1 |
| c.3809A>G | p.N1270S | 18 | P-domain | Missense | 0.000 | Damaging | 1.000 | Probably damaging | 3*PS, 1*PM, 2*PM, 3*PP | P | 1.69 | 2 | 40 |
| c.3818C>A | p.P1273Q | 18 | P-domain | Missense | 0.000 | Damaging | 1.000 | Probably damaging | 1*PM, 2*PM, 3*PP, 5*PP | LP | 0.08 | 0 | 2 |
| c.3818C>T | p.P1273L | 18 | P-domain | Missense | 0.000 | Damaging | 1.000 | Probably damaging | 3*PS, 2*PM, 3*PP, 5*PP | P | 0.08 | 0 | 2 |
| c.3832G>A | p.A1278T | 18 | P-domain | Missense | 0.001 | Damaging | 1.000 | Probably damaging | 1*PM, 2*PM, 3*PP | LP | 0.04 | 0 | 1 |
| c.3836A>G | p.D1279G | 18 | P-domain | Missense | 0.008 | Damaging | 0.999 | Probably damaging | 1*PM, 2*PM, 3*PP | LP | 0.19 | 0 | 5 |
| c.3839T>G | **p.M1280R** | **18** | **P-domain** | **Missense** | **0.000** | **Damaging** | **0.084** | **Benign** | **1*PM, 2*PM, 3*PP** | **LP** | **0.04** | **0** | **1** |
| c.3842G>A | p.G1281D | 18 | P-domain | Missense | 0.000 | Damaging | 1.000 | Probably damaging | 1*PM, 2*PM, 3*PP | LP | 0.12 | 0 | 3 |
| c.3843dup | p.V1282CfsX21 | 18 | P-domain | Frameshift | n.a | n.a | n.a | n.a | 1*PVS | P | 0.12 | 0 | 3 |
| c.3848C>T | p.A1283V | 18 | P-domain | Missense | 0.001 | Damaging | 1.000 | Probably damaging | 1*PM, 2*PM, 3*PP | LP | 0.08 | 0 | 2 |
| c.3851T>A | **p.I1284N** | **18** | **P-domain** | **Missense** | **0.000** | **Damaging** | **1.000** | **Probably damaging** | **1*PM, 2*PM, 3*PP** | **LP** | **0.04** | **0** | **1** |
| c.3854G>A | **p.G1285D** | **18** | **P-domain** | **Missense** | **0.001** | **Damaging** | **1.000** | **Probably damaging** | **1*PM, 2*PM, 3*PP** | **LP** | **0.04** | **0** | **1** |
| c.3859G>A | p.G1287S | 18 | P-domain | Missense | 0.000 | Damaging | 1.000 | Probably damaging | 1*PM, 2*PM, 3*PP | LP | 0.15 | 0 | 4 |
| c.3860G>A | **p.G1287D** | **18** | **P-domain** | **Missense** | **0.000** | **Damaging** | **1.000** | **Probably damaging** | **1*PM, 2*PM, 3*PP** | **LP** | **0.04** | **0** | **1** |
| c.3871G>A | p.A1291T | 18 | P-domain | Missense | 0.008 | Damaging | 0.995 | Probably damaging | 1*PM, 2*PM, 3*PP | LP | 0.04 | 0 | 1 |
| c.3877G>A | p.E1293K | 18 | P-domain | Missense | 0.001 | Damaging | 0.999 | Probably damaging | 1*PM, 2*PM, 3*PP | LP | 0.04 | 0 | 1 |
| c.3884C>T | p.A1295V | 18 | P-domain | Missense | 0.001 | Damaging | 1.000 | Probably damaging | 1*PM, 2*PM, 3*PP, 5*PP | LP | 0.81 | 0 | 21 |
| c.3892_3894del | p.V1298del | 18 | P-domain | Inframe del | n.a | n.a | n.a | n.a | 2*PM, 4*PM, 5*PP | LP | 0.04 | 0 | 1 |
| c.3896T>G | p.L1299R | 18 | P-domain | Missense | 0.000 | Damaging | 1.000 | Probably damaging | 1*PM, 2*PM, 3*PP | LP | 0.04 | 0 | 1 |
| c.3901dup | p.R1301KfsX3 | 18 | P-domain | Frameshift | n.a | n.a | n.a | n.a | 1*PVS | P | 0.12 | 0 | 3 |
| c.3903+1G>T | **n.a** | **18** | **P-domain** | **Splicing** | **n.a** | **n.a** | **n.a** | **n.a** | **1*PVS** | **P** | **0.04** | **0** | **1** |
| c.3903+2T>G | n.a | 18 | P-domain | Splicing | n.a | n.a | n.a | n.a | 1*PVS | P | 0.04 | 0 | 1 |
| c.3903+5G>A | n.a | 18 | P-domain | Splicing | n.a | n.a | n.a | n.a | n.a | LP | 0.04 | 0 | 1 |
| c.3904-3C>G | **n.a** | **19** | **TM7** | **Splicing** | **n.a** | **n.a** | **n.a** | **n.a** | **n.a** | **US** | **0.04** | **0** | **1** |
| c.3914T>C | p.L1305P | 19 | TM7 | Missense | 0.000 | Damaging | 1.000 | Probably damaging | 1*PM, 2*PM, 3*PP | LP | 0.08 | 0 | 2 |
| c.3917A>T | **p.D1306V** | **19** | **TM7** | **Missense** | **0.000** | **Damaging** | **1.000** | **Probably damaging** | **1*PM, 2*PM, 3*PP** | **LP** | **0.04** | **0** | **1** |
| c.3921del | **p.V1308WfsX21** | **19** | **TM7** | **Frameshift** | **n.a** | **n.a** | **n.a** | **n.a** | **1*PVS** | **P** | **0.04** | **0** | **1** |
| c.3932T>A | **p.I1311N** | **19** | **TM7** | **Missense** | **0.001** | **Damaging** | **1.000** | **Probably damaging** | **1*PM, 2*PM, 3*PP** | **LP** | **0.12** | **1** | **1** |
| c.3941C>G | p.S1314C | 19 | TM7 | Missense | 0.000 | Damaging | 1.000 | Probably damaging | 1*PM, 2*PM, 3*PP | LP | 0.04 | 0 | 1 |
| c.3955C>T | p.R1319X | 19 | TM7 | Nonsense | n.a | n.a | n.a | n.a | 1*PVS, 2*PM, 5*PP | P | 0.35 | 0 | 9 |
| c.3960G>C | p.R1320S | 19 | TM7 | Missense | 0.001 | Damaging | 1.000 | Probably damaging | 1*PM, 2*PM, 5*PP | LP | 0.08 | 0 | 2 |
| c.3971A>G | p.N1324S | 19 | TM7 | Missense | 0.000 | Damaging | 1.000 | Probably damaging | 1*PM, 2*PM, 3*PP | LP | 0.08 | 0 | 2 |
| c.3982G>A | p.A1328T | 19 | TM7 | Missense | 0.001 | Damaging | 1.000 | Probably damaging | 1*PM, 2*PM, 3*PP | LP | 0.23 | 0 | 6 |
| c.3983C>T | **p.A1328V** | **19** | **TM7** | **Missense** | **0.001** | **Damaging** | **1.000** | **Probably damaging** | **1*PM, 2*PM, 3*PP** | **LP** | **0.08** | **1** | **0** |
| c.4003G>C | p.G1335R | 19 | TM7 | Missense | 0.001 | Damaging | 1.000 | Probably damaging | 1*PM, 2*PM, 3*PP | LP | 0.19 | 0 | 5 |
| c.4006delA | p.I1336YfsX56 | 19 | TM7 | Frameshift | n.a | n.a | n.a | n.a | 1*PVS | P | 0.15 | 0 | 4 |
| c.4005_4006insTTATAATGGGTTGGG | p.G1335ins LXWVA | 19 | TM7 | Inframe ins | n.a | n.a | n.a | n.a | 2*PM, 4*PM, 5*PP | LP | 0.15 | 1 | 2 |
| c.4009C>G | **p.P1337A** | **19** | **TM7** | **Missense** | **0.000** | **Damaging** | **1.000** | **Probably damaging** | **1*PM, 2*PM, 3*PP** | **LP** | **0.04** | **0** | **1** |
| c.4014T>A | p.I1338I | 19 | TM7 | Splicing | n.a | n.a | n.a | n.a | 3*PS, 2*PM | P | 0.15 | 0 | 4 |
| c.4044_4054delinsGGATGGGCCCATCT | **p.1348_1352delins MDGPIS** | **20** | **TM8** | **Inframe ins** | **n.a** | **n.a** | **n.a** | **n.a** | **2*PM, 4*PM** | **LP** | **0.08** | **1** | **0** |
| c.4052_4070dup | **c.M1359LfsX24** | **20** | **TM8** | **Frameshift** | **n.a** | **n.a** | **n.a** | **n.a** | **1*PVS** | **P** | **0.04** | **0** | **1** |
| c.4063G>A | p.G1355S | 20 | TM8 | Missense | 0.100 | Damaging | 1.000 | Probably damaging | 1*PM, 2*PM, 3*PP | LP | 0.04 | 0 | 1 |
| c.4069G>A | **p.A1357T** | **20** | **TM8** | **Missense** | **0.004** | **Damaging** | **1.000** | **Probably damaging** | **1*PM, 2*PM, 3*PP** | **LP** | **0.04** | **0** | **1** |
| c.4072G>A | p.A1358T | 20 | TM8 | Missense | 0.001 | Damaging | 1.000 | Probably damaging | 1*PM, 2*PM, 3*PP | LP | 0.08 | 0 | 2 |
| c.4075A>G | p.M1359V | 20 | TM8 | Missense | 0.001 | Damaging | 1.000 | Probably damaging | 1*PM, 2*PM, 3*PP | LP | 0.08 | 0 | 2 |
| c.4109C>T | **p.S1370F** | **20** | **TM8** | **Missense** | **0.013** | **Damaging** | **1.000** | **Probably damaging** | **1*PM, 2*PM, 3*PP** | **LP** | **0.08** | **0** | **2** |
| c.4112T>C | p.L1371P | 20 | TM8 | Missense | 0.011 | Damaging | 1.000 | Probably damaging | 1*PM, 2*PM, 3*PP, 5*PP | LP | 0.15 | 1 | 2 |
| c.4114C>T | p.Q1372X | 20 | TM8 | Nonsense | n.a | n.a | n.a | n.a | 1*PVS, 2*PM, 3*PP, 5*PP | P | 0.96 | 0 | 25 |
| c.4120A>T | **p.K1374X** | **20** | **TM8** | **Nonsense** | **n.a** | **n.a** | **n.a** | **n.a** | **1*PVS, 2*PM, 3*PP** | **P** | **0.04** | **0** | **1** |
| c.4125-1G>C | **n.a** | **21** | **after TM8** | **Splicing** | **n.a** | **n.a** | **n.a** | **n.a** | **1*PVS** | **P** | **0.04** | **0** | **1** |
| c.4138G>C | **p.D1380H** | **21** | **after TM8** | **Missense** | **0.028** | **Damaging** | **0.724** | **Possibly damaging** | **1*PM, 2*PM, 3*PP** | **LP** | **0.04** | **0** | **1** |
| c.4144G>T | **p.E1382X** | **21** | **after TM8** | **Nonsense** | **n.a** | **n.a** | **n.a** | **n.a** | **1*PVS, 2*PM, 3*PP** | **P** | **0.04** | **0** | **1** |
| c.4176dup | **p.K1393EfsX15** | **21** | **after TM8** | **Frameshift** | **n.a** | **n.a** | **n.a** | **n.a** | **1*PVS** | **P** | **0.04** | **0** | **1** |
| c.4183dup | **p.L1395PfsX13** | **21** | **after TM8** | **Frameshift** | **n.a** | **n.a** | **n.a** | **n.a** | **1*PVS** | **P** | **0.08** | **0** | **2** |

Class.:classification; Freq.: frequency; Hom.: homozygous; Het.: heterozygous; n.a: not applicable; del: deletion; ins: insertion. Novel mutations are shown in boldface letters.

^a^<http://provean.jcvi.org/index.php>;

^b^<http://genetics.bwh.harvard.edu/pph2/index.shtml>;

^c^<http://wintervar.wglab.org/>.
