# Supplementary material for: Clinical and genetic characterization of a large cohort of patients with Wilson’s disease in China
Source: Transl Neurodegener. 2022 Feb 28;11:13. doi: 10.1186/s40035-022-00287-0 (PMC8883683; doi:10.1186/s40035-022-00287-0)
Supplement: Supplementary file 3 — Additional file 3: Table S3. Non-pathogenic variants of ATP7B identified in this study. [file 40035_2022_287_MOESM3_ESM.docx]

Table S3. Non-pathogenic variants of the ATP7B gene.

| Nucleotide mutation | Protein alteration | Location | Type | InterVar ^#^ |
| --- | --- | --- | --- | --- |
| c.-123_-119dup | - | 5’UTR | Duplication | 1*BA, 1*BS |
| c.-75C>A | - | 5’UTR | - | 1*BA, 1*BS |
| c.432G>A | **p.V144V** | **Exon 2** | **Silent** | **2*PM, 4*BP, 7*BP** |
| c.600T>C | **p.H200H** | **Exon 2** | **Silent** | **2*PM, 4*BP, 7*BP** |
| c.747G>A | p.L249L | Exon 2 | Silent | 2*PM, 4*BP, 7*BP |
| c.1216T>G | p.S406A | Exon 2 | Missense | 1*BA, 1*BS, 4*BP, 6*BP |
| c.1366G>C | p.L456V | Exon 3 | Missense | 1*BA, 1*BS, 4*BP, 6*BP |
| c.1544-53A>C | - | Intron 4 | - | **-** |
| c.1554C>T | p.S518S | Exon 4 | Silent | 2*PM, 4*BP, 7*BP |
| c.1869+9_+17del | **-** | **Intron 5** | **Deletion** | **-** |
| c.2145C>T | p.Y715Y | Exon 8 | Silent | 2*PM, 4*BP, 7*BP |
| c.2256G>A | **p.V752V** | **Exon 8** | **Silent** | **2*PM, 4*BP, 7*BP** |
| c.2292C>T | p.F764F | Exon 8 | Silent | 2*PM, 4*BP, 6*BP, 7*BP |
| c.2310C>G | p.L770L | Exon 8 | Silent | 2*PM, 4*BP, 6*BP, 7*BP |
| c.2495A>G | p.K832R | Exon 10 | Missense | 1*BA, 1*BS, 6*BP |
| c.2576-30A>G | - | Intron 10 | - | - |
| c.2576-6G>A | - | Intron 10 | - | - |
| c.2583C>T | p.A861A | Exon 11 | Silent | 2*PM, 4*BP, 6*BP, 7*BP |
| c.2785A>G | p.I929V | Exon 12 | Missense | 1*PM, 1*BS, 6*BP |
| c.2835C>T | p.I945I | Exon 12 | Silent | 2*PM, 4*PM, 7*BP |
| c.2855G>A | p.R952K | Exon 12 | Missense | 1*PM, 1*BA, 1*BS, 6*BP |
| c.2866-13G>C | - | Intron 12 | - | - |
| c.2973G>A | p.T991T | Exon 13 | Silent | 1*BA, 1*BS, 4* BP, 6*BP, 7*BP |
| c.3009G>A | p.A1003A | Exon 13 | Silent | 1*BS, 4* BP, 6*BP, 7*BP |
| c.3015C>T | p.N1005N | Exon 13 | Silent | 1*BS, 4* BP, 6*BP, 7*BP |
| c.3045G>A | p.L1015L | Exon 13 | Silent | 1*BS, 4* BP, 6*BP, 7*BP |
| c.3413-27G>C | - | Intron 18 | - | - |
| c.3419T>C | p.V1140A | Exon 16 | Missense | 1*PM, 1*BA, 1*BS, 4* BP, 6*BP |
| c.3426G>C | p.Q1142H | Exon 16 | Missense | 1*PM, 2*PM, 3*PP |
| c.3460C>T | p.L1154L | Exon 16 | Silent | 2*PM, 4*BP, 7*BP |
| c.3889G>A | p.V1297I | Exon 18 | Missense | 1*PM, 3*PP, 1*BS |
| c.3891C>T | p.V1297V | Exon 18 | Silent | 5*PP, 1*BS, 4*BP, 7*BP |
| c.3903+6C>T | - | Intron 18 | - | 1*BA, 1*BS |
| c.4021+50G>C | - | Intron 19 | - | 1*BA, 1*BS |

Novel mutations are shown in boldface letters.

^#^http://wintervar.wglab.org/
