# Supplementary material for: Clinical and genetic characterization of a large cohort of patients with Wilson’s disease in China
Source: Transl Neurodegener. 2022 Feb 28;11:13. doi: 10.1186/s40035-022-00287-0 (PMC8883683; doi:10.1186/s40035-022-00287-0)
Supplement: Supplementary file 4 — Additional file 4: Fig. S1. Sequencing results for 64 novel missense variants of the ATP7B gene. Fig. S2. Sequencing results for novel variants of the ATP7B gene except for missense variants. Fig. S3. Homology comparisons of novel missense variants in the ATP7B protein. [file 40035_2022_287_MOESM4_ESM.docx]

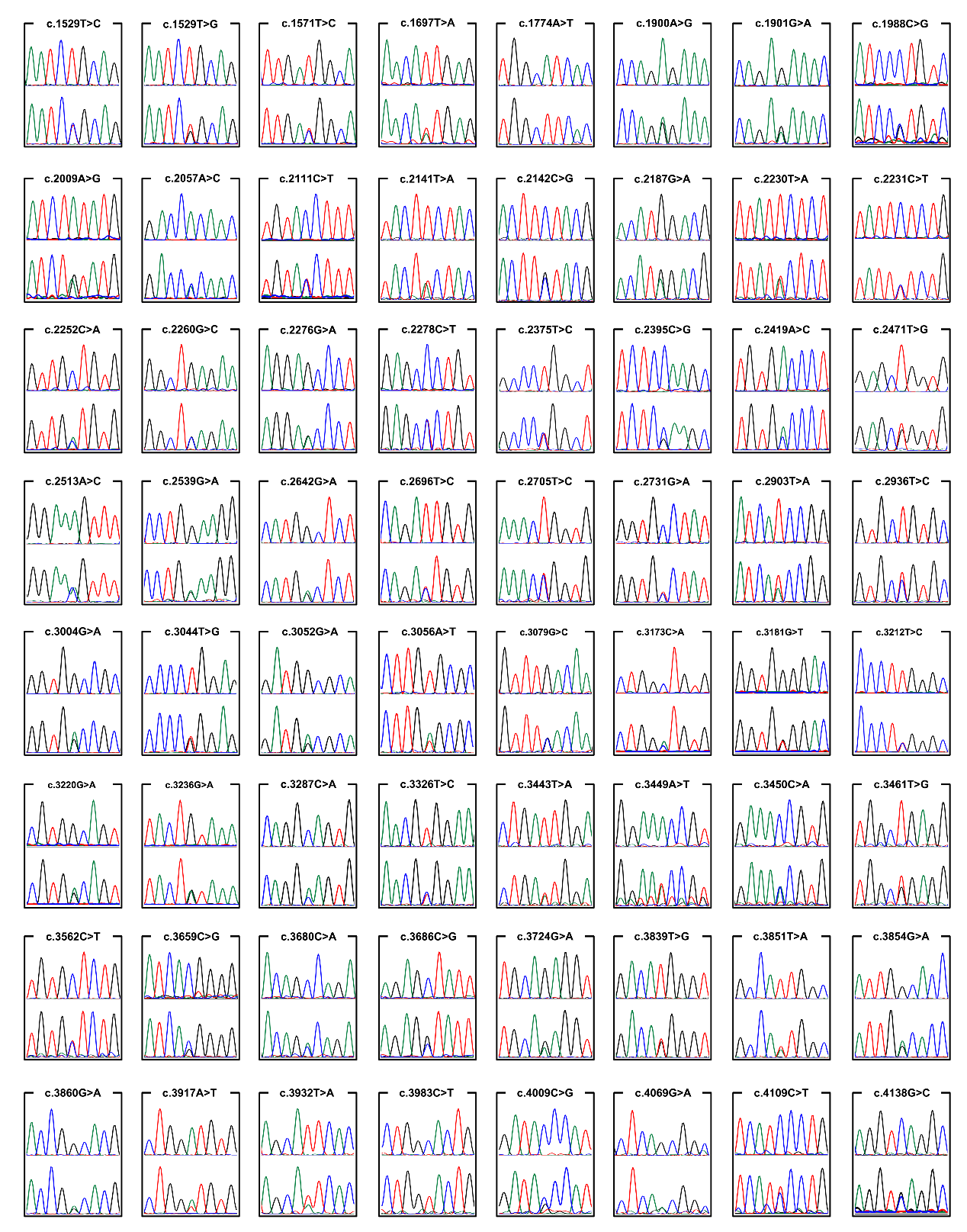


Figure S1. Sequencing results for 64 novel missense variants of the ATP7B gene. Both the normal sequences (upper chromatogram) and variants (lower chromatogram) are presented. The c.2731G>A, c.3056A>T, and c.4138G>C variants are shown in reverse sequence, and the others are illustrated in forward sequence.


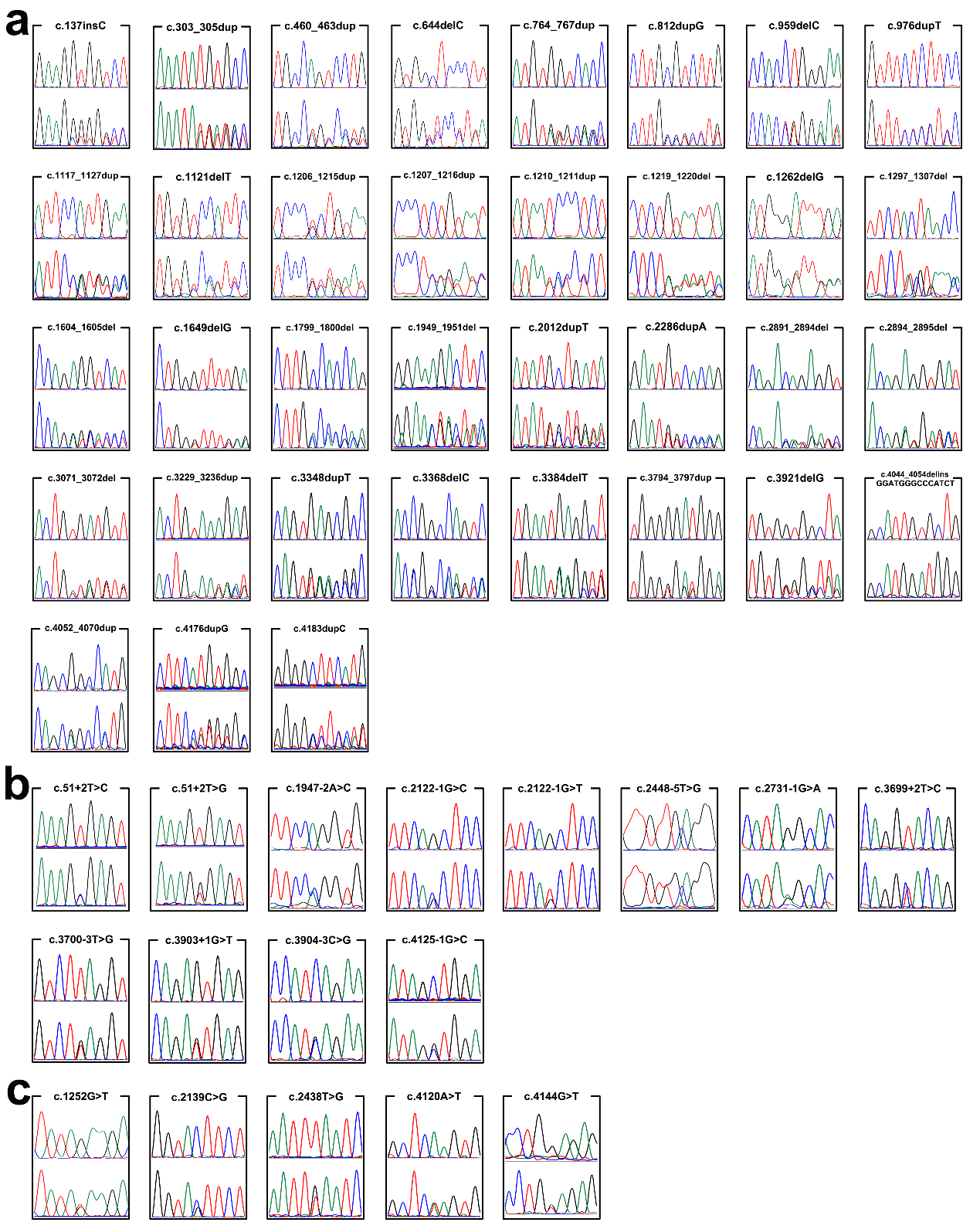


Figure S2. Sequencing results for novel variants of the ATP7B gene except for missense variants. (a) Small insertions and deletions; (b) Splice site variants; (c) Nonsense variants. Both the normal sequences (upper chromatogram) and variants (lower chromatogram) are presented. The c.1949_1951del, c.2286dupA, c.4176dupG, c.4183dupC, and c.4125-1G>C variants are shown in reverse sequence, and the others are shown in forward sequence.


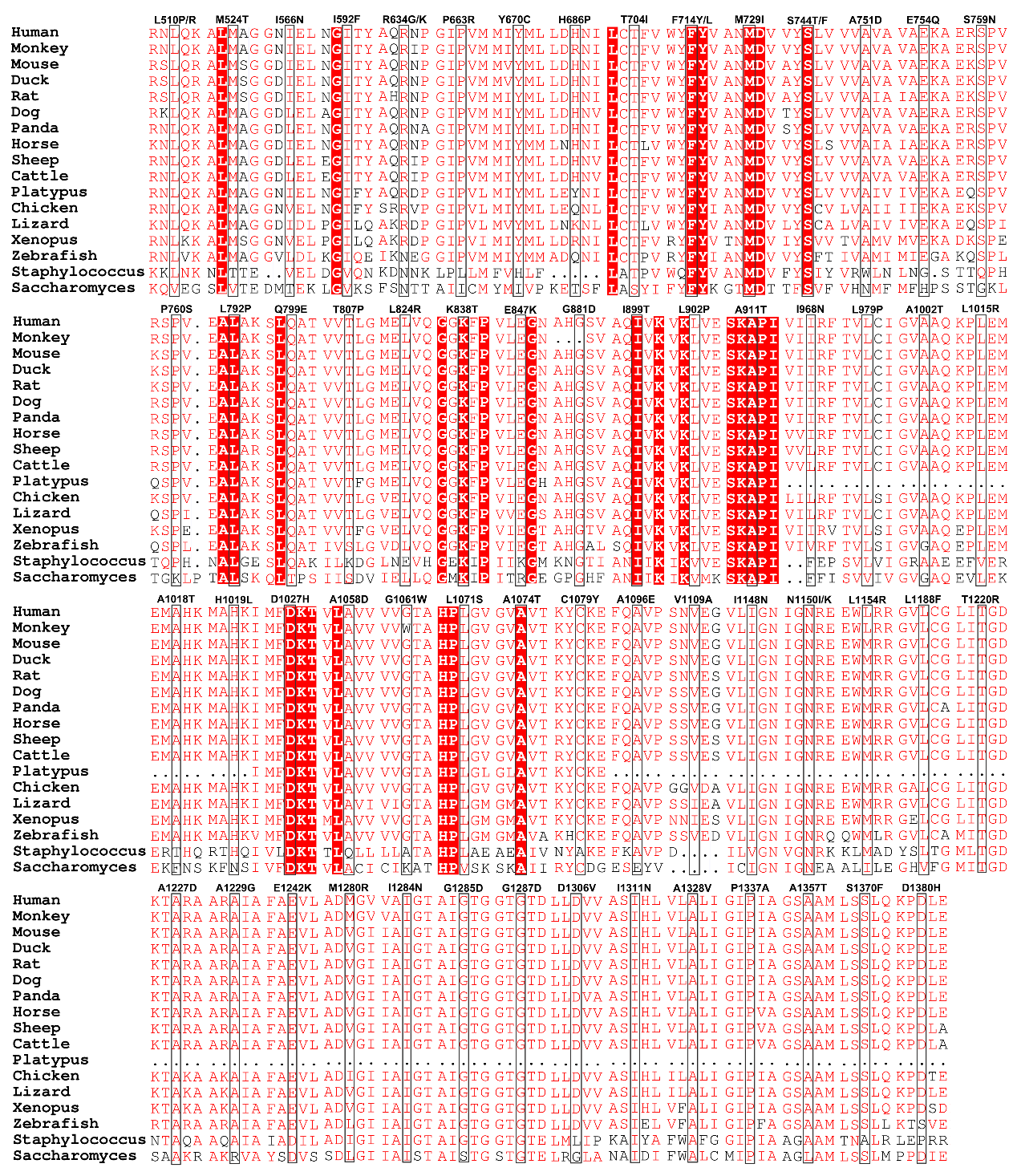


Figure S3. Homology comparisons of novel missense variants in the ATP7B protein. Conservation analysis of the ATP7B protein was performed with ENDscript server among 17 different species.
