# Supplementary material for: Clinical and genetic characterization of a large cohort of patients with Wilson’s disease in China
Source: Transl Neurodegener. 2022 Feb 28;11:13. doi: 10.1186/s40035-022-00287-0 (PMC8883683; doi:10.1186/s40035-022-00287-0)
Supplement: Supplementary file 5 — Additional file 5: Table S4. ATP7B variant combinations in this cohort. [file 40035_2022_287_MOESM5_ESM.doc]

Table S4. ATP7B variant combinations in this cohort.

| **Genotypes** | **N** | **Exon** | |
| --- | --- | --- | --- |
| c.2333G>T/? | 33 | 8 |  |
| c.2975C>T/? | 29 | 13 |  |
| c.2621C>T/? | 10 | 11 |  |
| c.2924C>A/? | 3 | 13 |  |
| c.3316G>A/? | 3 | 15 |  |
| c.4114C>T/? | 3 | 20 |  |
| c.1708-1G>C/c.1168A>G | 2 | 5 | 2 |
| c.1708-5T>G/? | 2 | 5 |  |
| c.3700del/? | 2 | 18 |  |
| c.3809A>G/? | 2 | 18 |  |
| c.268_270del/? | 1 | 2 |  |
| c.525dup/? | 1 | 2 |  |
| c.1470C>A/? | 1 | 3 |  |
| c.1543+1G>T/? | 1 | 3 |  |
| c.1988C>G/? | 1 | 7 |  |
| c.2122-1G>C/? | 1 | 8 |  |
| c.2128G>A/? | 1 | 8 |  |
| c.2276G>A/? | 1 | 8 |  |
| c.2304dup/? | 1 | 8 |  |
| c.2332C>T/? | 1 | 8 |  |
| c.2604del/? | 1 | 11 |  |
| c.2620G>C/? | 1 | 11 |  |
| c.2659del/? | 1 | 11 |  |
| c.2668G>A/? | 1 | 11 |  |
| c.2785A>G/? | 1 | 12 |  |
| c.2804C>T/? | 1 | 12 |  |
| c.2930C>T/? | 1 | 13 |  |
| c.3044T>C/? | 1 | 13 |  |
| c.3263T>C/? | 1 | 15 |  |
| c.3517G>A/? | 1 | 16 |  |
| c.3556G>A/? | 1 | 16 |  |
| c.3646G>A/? | 1 | 17 |  |
| c.3800A>G/? | 1 | 18 |  |
| c.3859G>A/? | 1 | 18 |  |
| c.3917A>T/? | 1 | 19 |  |
| c.3955C>T/? | 1 | 19 |  |
| c.4112T>C/? | 1 | 20 |  |

| **Genotypes** | **N** | **Exon** | | |
| --- | --- | --- | --- | --- |
| c.2333G>T/c.2333G>T | 125 | 8 | 8 |  |
| c.2333G>T/c.2975C>T | 90 | 8 | 13 |  |
| c.2333G>T/c.2621C>T | 53 | 8 | 11 |  |
| c.2975C>T/c.2975C>T | 39 | 13 | 13 |  |
| c.2333G>T/c.2755C>G | 25 | 8 | 12 |  |
| c.2333G>T/c.2924C>A | 16 | 8 | 13 |  |
| c.2975C>T/c.2621C>T | 16 | 13 | 11 |  |
| c.2333G>T/c.3316G>A | 13 | 8 | 15 |  |
| c.2333G>T/c.3646G>A | 12 | 8 | 17 |  |
| c.2333G>T/c.3443T>C | 11 | 8 | 16 |  |
| c.2333G>T/c.3517G>A | 10 | 8 | 16 |  |
| c.2333G>T/c.3809A>G | 9 | 8 | 18 |  |
| c.2333G>T/c.1708-1G>C | 9 | 8 | 5 |  |
| c.2333G>T/c.1708-5T>G | 9 | 8 | 5 |  |
| c.2333G>T/c.2294A>G | 8 | 8 | 8 |  |
| c.2333G>T/c.2304dup | 8 | 8 | 8 |  |
| c.2975C>T/c.2755C>G | 8 | 13 | 12 |  |
| c.2975C>T/c.3443T>C | 8 | 13 | 16 |  |
| c.2333G>T/c.2662A>C | 7 | 8 | 11 |  |
| c.2333G>T/c.4114C>T | 7 | 8 | 20 |  |
| c.2975C>T/c.2804C>T | 7 | 13 | 12 |  |
| c.2333G>T/c.2804C>T | 6 | 8 | 12 |  |
| c.2975C>T/c.3809A>G | 6 | 13 | 18 |  |
| c.2621C>T/c.2621C>T | 6 | 11 | 11 |  |
| c.2333G>T/c.2827G>A | 5 | 8 | 12 |  |
| c.2333G>T/c.525dup | 5 | 8 | 2 |  |
| c.2333G>T/c.994G>T | 5 | 8 | 2 |  |
| c.2975C>T/c.2304dup | 5 | 13 | 8 |  |
| c.2975C>T/c.3316G>A | 5 | 13 | 15 |  |
| c.2621C>T/c.3646G>A | 5 | 11 | 17 |  |
| c.2621C>T/c.2304dup | 5 | 11 | 8 |  |
| c.2333G>T/c.2620G>C | 4 | 8 | 11 |  |
| c.2333G>T/c.2668G>A | 4 | 8 | 11 |  |
| c.2333G>T/c.3263T>C | 4 | 8 | 15 |  |
| c.2333G>T/c.3700del | 4 | 8 | 18 |  |
| c.2975C>T/c.1708-1G>C | 4 | 13 | 5 |  |
| c.2975C>T/c.1708-5T>G | 4 | 13 | 5 |  |
| c.2975C>T/c.2294A>G | 4 | 13 | 8 |  |
| c.2975C>T/c.2924C>A | 4 | 13 | 13 |  |
| c.2975C>T/c.525dup | 4 | 13 | 2 |  |
| c.2975C>T/c.3982G>A | 4 | 13 | 19 |  |
| c.2333G>T/c.2333G>A | 3 | 8 | 8 |  |
| c.2333G>T/c.2790_2792del | 3 | 8 | 12 |  |
| c.2333G>T/c.2828G>A | 3 | 8 | 12 |  |
| c.2333G>T/c.3029A>C | 3 | 8 | 13 |  |
| c.2333G>T/c.3459G>T | 3 | 8 | 16 |  |
| c.2333G>T/c.3532A>G | 3 | 8 | 16 |  |
| c.2333G>T/c.3766_3767dup | 3 | 8 | 18 |  |
| c.2333G>T/c.3859G>A | 3 | 8 | 18 |  |
| c.2333G>T/c.3884C>T | 3 | 8 | 18 |  |
| c.2333G>T/c.1543+1G>T | 3 | 8 | 3 |  |
| c.2333G>T/c.2122-1G>C | 3 | 8 | 8 |  |
| c.2333G>T/c.2332C>T | 3 | 8 | 8 |  |
| c.2975C>T/c.2333G>A | 3 | 13 | 8 |  |
| c.2975C>T/c.2620G>C | 3 | 13 | 11 |  |
| c.2975C>T/c.2662A>C | 3 | 13 | 11 |  |
| c.2975C>T/c.3884C>T | 3 | 13 | 18 |  |
| c.2621C>T/c.2662A>C | 3 | 11 | 11 |  |
| c.2621C>T/c.3809A>G | 3 | 11 | 18 |  |
| c.2924C>A/c.2924C>A | 3 | 13 | 13 |  |
| c.3443T>C/c.3443T>C | 3 | 16 | 16 |  |
| c.3646G>A/c.3646G>A | 3 | 17 | 17 |  |
| c.2333G>T/c.2810del | 2 | 8 | 12 |  |
| c.2333G>T/c.2906G>A | 2 | 8 | 13 |  |
| c.2333G>T/c.2930C>T | 2 | 8 | 13 |  |
| c.2333G>T/c.2939G>A | 2 | 8 | 13 |  |
| c.2333G>T/c.3007G>A | 2 | 8 | 13 |  |
| c.2333G>T/c.3243G>A | 2 | 8 | 14 |  |
| c.2333G>T/c.3446G>A | 2 | 8 | 16 |  |
| c.2333G>T/c.4183dup | 2 | 8 | 21 |  |
| c.2333G>T/c.1470C>A | 2 | 8 | 3 |  |
| c.2333G>T/c.1531C>T | 2 | 8 | 3 |  |
| c.2333G>T/c.1846C>T | 2 | 8 | 5 |  |
| c.2333G>T/c.2128G>A | 2 | 8 | 8 |  |
| c.2333G>T/c.2145C>A | 2 | 8 | 8 |  |
| c.2333G>T/c.2293G>A | 2 | 8 | 8 |  |
| c.2975C>T/c.1470C>A | 2 | 13 | 3 |  |
| c.2975C>T/c.1543+1G>T | 2 | 13 | 3 |  |
| c.2975C>T/c.2145C>A | 2 | 13 | 8 |  |
| c.2975C>T/c.2187G>A | 2 | 13 | 8 |  |
| c.2975C>T/c.2356-2A＞G | 2 | 13 | 9 |  |
| c.2975C>T/c.2668G>A | 2 | 13 | 11 |  |
| c.2975C>T/c.994G>T | 2 | 13 | 2 |  |
| c.2975C>T/c.3517G>A | 2 | 13 | 16 |  |
| c.2621C>T/c.2755C>G | 2 | 11 | 12 |  |
| c.2621C>T/c.3089G>A | 2 | 11 | 14 |  |
| c.2621C>T/c.3377_3378del | 2 | 11 | 15 |  |
| c.2621C>T/c.3836A>G | 2 | 11 | 18 |  |
| c.2621C>T/c.3955C>T | 2 | 11 | 19 |  |
| c.2621C>T/c.2333G>A | 2 | 11 | 8 |  |
| c.2621C>T/c.2590_2593dup | 2 | 11 | 11 |  |
| c.2755C>G/c.2304dup | 2 | 12 | 8 |  |
| c.2755C>G/c.2332C>T | 2 | 12 | 8 |  |
| c.2755C>G/c.2336G>A | 2 | 12 | 8 |  |
| c.2755C>G/c.525dup | 2 | 12 | 2 |  |
| c.2755C>G/c.3443T>C | 2 | 12 | 16 |  |
| c.2755C>G/c.3809A>G | 2 | 12 | 18 |  |
| c.2304dup/c.2304dup | 2 | 8 | 8 |  |
| c.1708-1G>C/c.2294A>G | 2 | 5 | 8 |  |
| c.2620G>C/c.2620G>C | 2 | 11 | 11 |  |
| c.2804C>T/c.2810del | 2 | 12 | 12 |  |
| c.2662A>C/c.3316G>A | 2 | 11 | 15 |  |
| c.2790_2792del/c.3316G>A | 2 | 12 | 15 |  |
| c.2790_2792del/c.3443T>C | 2 | 12 | 16 |  |
| c.3316G>A/c.3517G>A | 2 | 15 | 16 |  |
| c.3443T>C/c.3646G>A | 2 | 16 | 17 |  |
| c.3316G>A/c.3700del | 2 | 15 | 18 |  |
| c.2294A>G/c.3809A>G | 2 | 8 | 18 |  |
| c.3316G>A/c.3809A>G | 2 | 15 | 18 |  |
| c.3646G>A/c.3809A>G | 2 | 17 | 18 |  |
| c.3809A>G/c.3809A>G | 2 | 18 | 18 |  |
| c.525dup/c.3884C>T | 2 | 2 | 18 |  |
| c.994G>T/c.994G>T | 2 | 2 | 2 |  |
| c.2333G>T/c.2336G>A | 1 | 8 | 8 |  |
| c.2333G>T/c.2356-2A＞G | 1 | 8 | 9 |  |
| c.2333G>T/c.2447+5G>T | 1 | 8 | 9 |  |
| c.2333G>T/c.2455C>T | 1 | 8 | 10 |  |
| c.2333G>T/c.2471T>G | 1 | 8 | 10 |  |
| c.2333G>T/c.2549C>T | 1 | 8 | 10 |  |
| c.2333G>T/c.2590_2593dup | 1 | 8 | 11 |  |
| c.2333G>T/c.2605G>A | 1 | 8 | 11 |  |
| c.2333G>T/c.2642G>A | 1 | 8 | 11 |  |
| c.2333G>T/c.2659del | 1 | 8 | 11 |  |
| c.2333G>T/c.2696T>C | 1 | 8 | 11 |  |
| c.2333G>T/c.2697_2723del | 1 | 8 | 11 |  |
| c.2333G>T/c.2752G>A | 1 | 8 | 12 |  |
| c.2333G>T/c.2806_2808del | 1 | 8 | 12 |  |
| c.2333G>T/c.2903T>A | 1 | 8 | 13 |  |
| c.2333G>T/c.2905C>T | 1 | 8 | 13 |  |
| c.2333G>T/c.2957C>T | 1 | 8 | 13 |  |
| c.2333G>T/c.2963G>T | 1 | 8 | 13 |  |
| c.2333G>T/c.2998G>A | 1 | 8 | 13 |  |
| c.2333G>T/c.3028A>G | 1 | 8 | 13 |  |
| c.2333G>T/c.3044T>C | 1 | 8 | 13 |  |
| c.2333G>T/c.3089G>A | 1 | 8 | 14 |  |
| c.2333G>T/c.3104G>T | 1 | 8 | 14 |  |
| c.2333G>T/c.3243+5G>A | 1 | 8 | 14 |  |
| c.2333G>T/c.3244-2A>G | 1 | 8 | 15 |  |
| c.2333G>T/c.3443T>A | 1 | 8 | 16 |  |
| c.2333G>T /c.303_305dup/c.3461T>G | 1 | 8 | 2 | 16 |
| c.2333G>T/c.3557-2A>G | 1 | 8 | 17 |  |
| c.2333G>T/c.3562C>T | 1 | 8 | 17 |  |
| c.2333G>T/c.3587A>G | 1 | 8 | 17 |  |
| c.2333G>T/c.3659C>G | 1 | 8 | 17 |  |
| c.2333G>T/c.3662_3664del | 1 | 8 | 17 |  |
| c.2333G>T/c.3686C>G | 1 | 8 | 17 |  |
| c.2333G>T/c.3700-1G>A | 1 | 8 | 18 |  |
| c.2333G>T/c.3818C>T | 1 | 8 | 18 |  |
| c.2333G>T/c.3839T>G | 1 | 8 | 18 |  |
| c.2333G>T/c.3848C>T | 1 | 8 | 18 |  |
| c.2333G>T/c.3851T>A | 1 | 8 | 18 |  |
| c.2333G>T/c.3854G>A | 1 | 8 | 18 |  |
| c.2333G>T/c.3860G>A | 1 | 8 | 18 |  |
| c.2333G>T/c.3877G>A | 1 | 8 | 18 |  |
| c.2333G>T/c.3892_3894del | 1 | 8 | 18 |  |
| c.2333G>T/c.3896T>G | 1 | 8 | 18 |  |
| c.2333G>T/c.3914T>C | 1 | 8 | 19 |  |
| c.2333G>T/c.3921del | 1 | 8 | 19 |  |
| c.2333G>T/c.3941C>G | 1 | 8 | 19 |  |
| c.2333G>T/c.3955C>T | 1 | 8 | 19 |  |
| c.2333G>T/c.3960G>C | 1 | 8 | 19 |  |
| c.2333G>T/c.4003G>C | 1 | 8 | 19 |  |
| c.2333G>T/c.4005_4006insTTATAATGGGTTGGG | 1 | 8 | 19 |  |
| c.2333G>T/c.4009C>G | 1 | 8 | 19 |  |
| c.2333G>T/c.4014T>A | 1 | 8 | 19 |  |
| c.2333G>T/c.4052_4070dup | 1 | 8 | 20 |  |
| c.2333G>T/c.4063G>A | 1 | 8 | 20 |  |
| c.2333G>T/c.4072G>A | 1 | 8 | 20 |  |
| c.2333G>T/c.4109C>T | 1 | 8 | 20 |  |
| c.2333G>T/c.4120A>T | 1 | 8 | 20 |  |
| c.2333G>T/c.4138G>C | 1 | 8 | 21 |  |
| c.2333G>T/c.1207_1216dup | 1 | 8 | 2 |  |
| c.2333G>T/c.1162C>T | 1 | 8 | 2 |  |
| c.2333G>T/c.122A>G | 1 | 8 | 2 |  |
| c.2333G>T/c.1262del | 1 | 8 | 2 |  |
| c.2333G>T/c.1571T>C | 1 | 8 | 4 |  |
| c.2333G>T/c.1586A>G | 1 | 8 | 4 |  |
| c.2333G>T/c.1648_1654del | 1 | 8 | 4 |  |
| c.2333G>T/c.1772G>A | 1 | 8 | 5 |  |
| c.2333G>T/c.1817T>G | 1 | 8 | 5 |  |
| c.2333G>T/c.2009A>G | 1 | 8 | 7 |  |
| c.2333G>T/c.2012dup | 1 | 8 | 7 |  |
| c.2333G>T/c.2075T>C | 1 | 8 | 7 |  |
| c.2333G>T/c.2078C>G | 1 | 8 | 7 |  |
| c.2333G>T/c.2139C>G | 1 | 8 | 8 |  |
| c.2333G>T/c.2230T>C | 1 | 8 | 8 |  |
| c.2333G>T/c.2252C>A | 1 | 8 | 8 |  |
| c.2333G>T/c.2260G>C | 1 | 8 | 8 |  |
| c.2333G>T/c.2308C>T | 1 | 8 | 8 |  |
| c.2333G>T/c.314C>A | 1 | 8 | 2 |  |
| c.2333G>T/c.650T>G | 1 | 8 | 2 |  |
| c.2333G>T/c.775del | 1 | 8 | 2 |  |
| c.2333G>T/c.812dup | 1 | 8 | 2 |  |
| c.2333G>T/c.959del | 1 | 8 | 2 |  |
| c.2333G>T/c.970A>T | 1 | 8 | 2 |  |
| c.2333G>T/c.976dup | 1 | 8 | 2 |  |
| c.2975C>T/c.1219_1220del | 1 | 13 | 2 |  |
| c.2975C>T/c.1403_1416del | 1 | 13 | 3 |  |
| c.2975C>T/c.1475T>C | 1 | 13 | 3 |  |
| c.2975C>T/c.1697T>A | 1 | 13 | 4 |  |
| c.2975C>T/c.1707+5G>A | 1 | 13 | 4 |  |
| c.2975C>T/c.1803del | 1 | 13 | 5 |  |
| c.2975C>T/c.1847G>A | 1 | 13 | 5 |  |
| c.2975C>T/c.1947-2A>C | 1 | 13 | 7 |  |
| c.2975C>T/c.2009_2012dup | 1 | 13 | 7 |  |
| c.2975C>T/c.2078C>G | 1 | 13 | 7 |  |
| c.2975C>T/c.2120A>G | 1 | 13 | 7 |  |
| c.2975C>T/c.2122-1G>T | 1 | 13 | 8 |  |
| c.2975C>T/c.2252C>A | 1 | 13 | 8 |  |
| c.2975C>T/c.2297C>T | 1 | 13 | 8 |  |
| c.2975C>T/c.2305A>G | 1 | 13 | 8 |  |
| c.2975C>T/c.2308C>T | 1 | 13 | 8 |  |
| c.2975C>T/c.2383C>T | 1 | 13 | 9 |  |
| c.2975C>T/c.2447+5G>T | 1 | 13 | 9 |  |
| c.2975C>T/c.2455C>T | 1 | 13 | 10 |  |
| c.2975C>T/c.2539G>A | 1 | 13 | 10 |  |
| c.2975C>T/c.2549C>T | 1 | 13 | 10 |  |
| c.2975C>T/c.2659del | 1 | 13 | 11 |  |
| c.2975C>T/c.2697_2723del | 1 | 13 | 11 |  |
| c.2975C>T/c.2731-1G>A | 1 | 13 | 12 |  |
| c.2975C>T/c.2790_2792del | 1 | 13 | 12 |  |
| c.2975C>T/c.2827G>A | 1 | 13 | 12 |  |
| c.2975C>T/c.2828G>A | 1 | 13 | 12 |  |
| c.2975C>T/c.2930C>T | 1 | 13 | 13 |  |
| c.2975C>T/c.2938T>C | 1 | 13 | 13 |  |
| c.2975C>T/c.2963G>T | 1 | 13 | 13 |  |
| c.2975C>T/c.51+2T>G | 1 | 13 | 1 |  |
| c.2975C>T/c.3007G>A | 1 | 13 | 13 |  |
| c.2975C>T/c.3028A>G | 1 | 13 | 13 |  |
| c.2975C>T/c.3089G>A | 1 | 13 | 14 |  |
| c.2975C>T/c.3140A>T | 1 | 13 | 14 |  |
| c.2975C>T/c.3155C>T | 1 | 13 | 14 |  |
| c.2975C>T/c.3263T>C | 1 | 13 | 15 |  |
| c.2975C>T/c.3310T>C | 1 | 13 | 15 |  |
| c.2975C>T/c.3348dup | 1 | 13 | 15 |  |
| c.2975C＞T/c.3368del | 1 | 13 | 15 |  |
| c.2975C>T/c.3446G>A | 1 | 13 | 16 |  |
| c.2975C>T/c.3451C>T | 1 | 13 | 16 |  |
| c.2975C>T/c.3452G>A | 1 | 13 | 16 |  |
| c.2975C>T/c.3459G>T | 1 | 13 | 16 |  |
| c.2975C>T/c.3562C>T | 1 | 13 | 17 |  |
| c.2975C>T/c.3646G>A | 1 | 13 | 17 |  |
| c.2975C>T/c.3871G>A | 1 | 13 | 18 |  |
| c.2975C>T/c.3901dup | 1 | 13 | 18 |  |
| c.2975C>T/c.3955C>T | 1 | 13 | 19 |  |
| c.2975C>T/c.3971A>G | 1 | 13 | 19 |  |
| c.2975C>T/c.4006del | 1 | 13 | 19 |  |
| c.2975C>T/c.4014T>A | 1 | 13 | 19 |  |
| c.2975C>T/c.4112T>C | 1 | 13 | 20 |  |
| c.2975C>T/c.4114C>T | 1 | 13 | 20 |  |
| c.2975C>T/c.4125-1G>C | 1 | 13 | 21 |  |
| c.2975C>T/c.4144G>T | 1 | 13 | 21 |  |
| c.2621C>T/c.2705T>C | 1 | 11 | 11 |  |
| c.2621C>T/c.2790_2792del | 1 | 11 | 12 |  |
| c.2621C>T/c.2795C>A | 1 | 11 | 12 |  |
| c.2621C>T/c.2828G>A | 1 | 11 | 12 |  |
| c.2621C>T/c.2924C>A | 1 | 11 | 13 |  |
| c.2621C>T/c.2963G>T | 1 | 11 | 13 |  |
| c.2621C>T/c.3028A>G | 1 | 11 | 13 |  |
| c.2621C>T/c.3271T>C | 1 | 11 | 15 |  |
| c.2621C>T/c.3310T>C | 1 | 11 | 15 |  |
| c.2621C>T/c.3316G>A | 1 | 11 | 15 |  |
| c.2621C>T/c.3384del | 1 | 11 | 15 |  |
| c.2621C>T/c.3443T>C | 1 | 11 | 16 |  |
| c.2621C>T/c.3459G>T | 1 | 11 | 16 |  |
| c.2621C>T/c.3517G>A | 1 | 11 | 16 |  |
| c.2621C>T/c.3532A>G | 1 | 11 | 16 |  |
| c.2621C>T/c.3587A>G | 1 | 11 | 17 |  |
| c.2621C>T/c.3662_3664del | 1 | 11 | 17 |  |
| c.2621C>T/c.3700del | 1 | 11 | 18 |  |
| c.2621C>T/c.3707T>C | 1 | 11 | 18 |  |
| c.2621C>T/c.3842G>A | 1 | 11 | 18 |  |
| c.2621C>T/c.3884C>T | 1 | 11 | 18 |  |
| c.2621C>T/c.4006del | 1 | 11 | 19 |  |
| c.2621C>T/c.4114C>T | 1 | 11 | 20 |  |
| c.2621C>T/c.1708-1G>C | 1 | 11 | 5 |  |
| c.2621C>T/c.1708-5T>G | 1 | 11 | 5 |  |
| c.2621C>T/c.1799_1800del | 1 | 11 | 5 |  |
| c.2621C>T/c.1803del | 1 | 11 | 5 |  |
| c.2621C>T/c.1846C>T | 1 | 11 | 5 |  |
| c.2621C>T/c.2165dup | 1 | 11 | 8 |  |
| c.2621C>T/c.2195T>C | 1 | 11 | 8 |  |
| c.2621C>T/c.2375T>C | 1 | 11 | 9 |  |
| c.2621C>T/c.2448-5T>G | 1 | 11 | 10 |  |
| c.2621C>T/c.2604del | 1 | 11 | 11 |  |
| c.2621C>T/c.2620G>C | 1 | 11 | 11 |  |
| c.2755C>G/c.1543+1G>T | 1 | 12 | 3 |  |
| c.2755C>G/c.1543+4A>G | 1 | 12 | 3 |  |
| c.2755C>G/c.1708-5T>G | 1 | 12 | 5 |  |
| c.2755C>G/c.1846C>T | 1 | 12 | 5 |  |
| c.2755C>G/c.2122-1G>T | 1 | 12 | 8 |  |
| c.2755C>G/c.2252C>A | 1 | 12 | 8 |  |
| c.2755C>G/c.2438T>G | 1 | 12 | 9 |  |
| c.2755C>G/c.2513A>C | 1 | 12 | 10 |  |
| c.2755C>G/c.3699+2T>C | 1 | 12 | 17 |  |
| c.2755C>G/c.764_767dup | 1 | 12 | 2 |  |
| c.2755C>G/c.2828G>A | 1 | 12 | 12 |  |
| c.2755C>G/c.2887C>T | 1 | 12 | 13 |  |
| c.2755C>G/c.3244-2A>G | 1 | 12 | 15 |  |
| c.2755C>G/c.3446G>A | 1 | 12 | 16 |  |
| c.2755C>G/c.3700del | 1 | 12 | 18 |  |
| c.2755C>G/c.3901dup | 1 | 12 | 18 |  |
| c.2755C>G/c.3914T>C | 1 | 12 | 19 |  |
| c.2304dup/c.2332C>T | 1 | 8 | 8 |  |
| c.2304dup/c.2662A>C | 1 | 8 | 11 |  |
| c.2304dup/c.2668G>A | 1 | 8 | 11 |  |
| c.2304dup/c.2697_2723del | 1 | 8 | 11 |  |
| c.2304dup/c.2804C>T | 1 | 8 | 12 |  |
| c.2304dup/c.2924C>A | 1 | 8 | 13 |  |
| c.2304dup/c.3053C>T | 1 | 8 | 13 |  |
| c.2304dup/c.3446G>A | 1 | 8 | 16 |  |
| c.2304dup/c.3505A>G | 1 | 8 | 16 |  |
| c.2304dup/c.3646G>A | 1 | 8 | 17 |  |
| c.2304dup/c.3955C>T | 1 | 8 | 19 |  |
| c.2304dup/ c.3244-2A>G/c.1426G>A | 1 | 8 | 15 | 3 |
| c.2304dup/c.1517_1518del | 1 | 8 | 3 |  |
| c.2304dup/c.1531C>T | 1 | 8 | 3 |  |
| c.2304dup/c.1649del | 1 | 8 | 4 |  |
| c.2304dup/c.1803del | 1 | 8 | 5 |  |
| c.2304dup/c.2145C>A | 1 | 8 | 8 |  |
| c.2304dup/c.2293G>A | 1 | 8 | 8 |  |
| c.2304dup/c.525dup | 1 | 8 | 2 |  |
| c.2621C>T/c.1449_1456del | 1 | 11 | 3 |  |
| c.994G>T/c.1529T>C | 1 | 2 | 3 |  |
| c.1531C>T/c.1531C>T | 1 | 3 | 3 |  |
| c.1543+1G>T/c.1604_1605del | 1 | 3 | 4 |  |
| c.1708-1G>C/c.1708-1G>C | 1 | 5 | 5 |  |
| c.1708-5T>G/c.1708-1G>C | 1 | 5 | 5 |  |
| c.1708-5T>G/c.1708-5T>G | 1 | 5 | 5 |  |
| c.1774A>T/c.1774A>T | 1 | 5 | 5 |  |
| c.1708-1G>C/c.1988C>G/c.1168A>G | 1 | 5 | 7 | 2 |
| c.1449_1456del/c.2057A>C | 1 | 3 | 7 |  |
| c.51+2T>G/c.2141T>A | 1 | 1 | 8 |  |
| c.460_463dup/c.2142C>G | 1 | 2 | 8 |  |
| c.122A>G/c.2145C>A | 1 | 2 | 8 |  |
| c.2145C>A/c.2145C>A | 1 | 8 | 8 |  |
| c.1543+1G>T/c.2261A>G | 1 | 3 | 8 |  |
| c.525dup/c.2267C>G | 1 | 2 | 8 |  |
| c.2267C>G/c.2286dup | 1 | 8 | 8 |  |
| c.525dup/c.2293G>A | 1 | 2 | 8 |  |
| c.1817T>G/c.2395C>G | 1 | 5 | 9 |  |
| c.2561A>T/c.2561A>T | 1 | 10 | 10 |  |
| c.2604del/c.2604del | 1 | 11 | 11 |  |
| c.1901G>A/c.2620G>C | 1 | 6 | 11 |  |
| c.1252G>T/c.2662A>C | 1 | 2 | 11 |  |
| c.1708-1G>C/c.2662A>C | 1 | 5 | 11 |  |
| c.2662A>C/c.2662A>C | 1 | 11 | 11 |  |
| c.51+2T>G/c.2662A>C | 1 | 1 | 11 |  |
| c.2668G>A/c.2668G>A | 1 | 11 | 11 |  |
| c.1121del/c.2731G>A | 1 | 2 | 12 |  |
| c.2294A>G/c.2752G>A | 1 | 8 | 12 |  |
| c.1708-1G>C/c.2790_2792del | 1 | 5 | 12 |  |
| c.2668G>A/c.2790_2792del | 1 | 11 | 12 |  |
| c.51+2T>G/c.2790_2792del | 1 | 1 | 12 |  |
| c.1531C>T/c.2804C>T | 1 | 3 | 12 |  |
| c.2620G>C/c.2804C>T | 1 | 11 | 12 |  |
| c.2337G>A/c.2827G>A | 1 | 8 | 12 |  |
| c.2827G>A/c.2827G>A | 1 | 12 | 12 |  |
| c.994G>T/c.2827G>A | 1 | 2 | 12 |  |
| c.1708-1G>C/c.2828G>A | 1 | 5 | 12 |  |
| c.1708-5T>G/c.2828G>A | 1 | 5 | 12 |  |
| c.2252C>A/c.2828G>A | 1 | 8 | 12 |  |
| c.2447+5G>T/c.2891_2894del | 1 | 9 | 13 |  |
| c.2447+5G>T/c.2906G>A | 1 | 9 | 13 |  |
| c.1708-5T>G/c.2924C>A | 1 | 5 | 13 |  |
| c.2419A>C/c.2924C>A | 1 | 9 | 13 |  |
| c.254G>T/c.2924C>A | 1 | 10 | 13 |  |
| c.51+1G>A/c.2924C>A | 1 | 1 | 13 |  |
| c.2659del/c.2963G>T | 1 | 11 | 13 |  |
| c.2924C>A/c.2963G>T | 1 | 13 | 13 |  |
| c.2120A>G/c.3004G>A | 1 | 7 | 13 |  |
| c.2620G>C/c.3028A>G | 1 | 11 | 13 |  |
| c.2804C>T/c.3028A>G | 1 | 12 | 13 |  |
| c.2195T>C/c.3044T>C | 1 | 8 | 13 |  |
| c.2293G>A/c.3044T>C | 1 | 8 | 13 |  |
| c.1708-1G>C/c.3044T>G/ c.1168A>G | 1 | 5 | 13 | 2 |
| c.1210_1211dup/c.3052G>A | 1 | 2 | 13 |  |
| c.2294A>G/c.3053C>T | 1 | 8 | 13 |  |
| c.2930C>T/c.3053C>T | 1 | 13 | 13 |  |
| c.1708-1G>C/c.3056A>C | 1 | 5 | 13 |  |
| c.2185A>G/c.3060+5G>T | 1 | 8 | 13 |  |
| c.3061-3C>A/c.3061-3C>A | 1 | 14 | 14 |  |
| c.2804C>T/c.3071_3072del | 1 | 12 | 14 |  |
| c.1901G>A/c.3079G>C | 1 | 6 | 14 |  |
| c.2327T>C/c.3089G>A | 1 | 8 | 14 |  |
| c.2924C>A/c.3104G>T | 1 | 13 | 14 |  |
| c.314C>A/c.3121C>T | 1 | 2 | 14 |  |
| c.2998G>A/c.3140A>T | 1 | 13 | 14 |  |
| c.3140A>T/c.3140A>T | 1 | 14 | 14 |  |
| c.1543+1G>T/c.3173C>A | 1 | 3 | 14 |  |
| c.2906G>A/c.3181G>T | 1 | 13 | 14 |  |
| c.525dup/c.3212T>C | 1 | 2 | 14 |  |
| c.2894_2895del/c.3220G>A | 1 | 13 | 14 |  |
| c.3140A>T/c.3229_3236dup | 1 | 14 | 14 |  |
| c.2111C>T/c.3244-2A>G | 1 | 7 | 15 |  |
| c.2828G>A/c.3244-2A>G | 1 | 12 | 15 |  |
| c.2936T>C/c.3274A>C | 1 | 13 | 15 |  |
| c.1470C>A/c.3316G>A | 1 | 3 | 15 |  |
| c.1595A>G/c.3316G>A | 1 | 4 | 15 |  |
| c.1988C>G/c.3316G>A | 1 | 7 | 15 |  |
| c.2038C>T/c.3316G>A | 1 | 7 | 15 |  |
| c.2938T>C/c.3316G＞A | 1 | 13 | 15 |  |
| c.2336G>A/c.3377_3378del | 1 | 8 | 15 |  |
| c.2356-2A＞G/c.3424C＞T | 1 | 9 | 16 |  |
| c.137ins/c.3443T>C | 1 | 2 | 16 |  |
| c.1949_1951del/c.3443T>C | 1 | 7 | 16 |  |
| c.2513A>C/c.3443T>C | 1 | 10 | 16 |  |
| c.2804C>T/c.3443T>C | 1 | 12 | 16 |  |
| c.3008C>T/c.3443T>C | 1 | 13 | 16 |  |
| c.3089G>A/c.3443T>C | 1 | 14 | 16 |  |
| c.314C>A/c.3443T>C | 1 | 2 | 16 |  |
| c.3809A>G/c.3443T>C | 1 | 18 | 16 |  |
| c.525dup/c.3443T>C | 1 | 2 | 16 |  |
| c.1900A>G/c.3446G>A | 1 | 6 | 16 |  |
| c.3818C>T/c.3449A>T | 1 | 18 | 16 |  |
| c.2894_2895del/c.3451C>T | 1 | 13 | 16 |  |
| c.3236G>A/c.3459G>T | 1 | 14 | 16 |  |
| c.2332C>T/c.3505A>G | 1 | 8 | 16 |  |
| c.1449_1456del/c.3517G>A | 1 | 3 | 16 |  |
| c.1803del/c.3517G>A | 1 | 5 | 16 |  |
| c.2231C>T/c.3517G>A | 1 | 8 | 16 |  |
| c.2827G>A/c.3517G>A | 1 | 12 | 16 |  |
| c.2333G>A/c.3532A>G | 1 | 8 | 16 |  |
| c.2327T>C/c.3551T>C | 1 | 8 | 16 |  |
| c.2827G>A/c.3562C>T | 1 | 12 | 17 |  |
| c.3443T>C/c.3562C>T | 1 | 16 | 17 |  |
| c.1117_1127dup/c.3646G>A | 1 | 2 | 17 |  |
| c.122A>G/c.3646G>A | 1 | 2 | 17 |  |
| c.1297_1307del/c.3646G>A | 1 | 3 | 17 |  |
| c.1449_1456del/c.3646G>A | 1 | 3 | 17 |  |
| c.1520_1523del/c.3646G>A | 1 | 3 | 17 |  |
| c.1571T>C/c.3646G>A | 1 | 4 | 17 |  |
| c.1708-1G>C/c.3646G>A | 1 | 5 | 17 |  |
| c.1745_1746del/c.3646G>A | 1 | 5 | 17 |  |
| c.1817T>G/c.3646G>A | 1 | 5 | 17 |  |
| c.2145C>A/c.3646G>A | 1 | 8 | 17 |  |
| c.2294A>G/c.3646G>A | 1 | 8 | 17 |  |
| c.2333G>A/c.3646G>A | 1 | 8 | 17 |  |
| c.2447+5G>T/c.3646G>A | 1 | 9 | 17 |  |
| c.3121C>T/c.3646G>A | 1 | 14 | 17 |  |
| c.3263T>C/c.3646G>A | 1 | 15 | 17 |  |
| c.3287C>A/c.3646G>A | 1 | 15 | 17 |  |
| c.3517G>A/c.3646G>A | 1 | 16 | 17 |  |
| c.3459G>T/c.3662_3664del | 1 | 16 | 17 |  |
| c.3316G>A/c.3700-3T>G | 1 | 15 | 18 |  |
| c.3316G>A /c.3700del/c.1529T>G | 1 | 15 | 18 | 3 |
| c.3680C>A/c.3700del | 1 | 17 | 18 |  |
| c.1449_1556del/c.3700del | 1 | 3 | 18 |  |
| c.1707+5G>A/c.3700del | 1 | 4 | 18 |  |
| c.1803del/c.3700del | 1 | 5 | 18 |  |
| c.2924C>A/c.3700del | 1 | 13 | 18 |  |
| c.2332C>T/c.3715G>T | 1 | 8 | 18 |  |
| c.2294A>G/c.3724G>A | 1 | 8 | 18 |  |
| c.3646G>A/c.3733C>G | 1 | 17 | 18 |  |
| c.2827G>A/c.3744G>C | 1 | 12 | 18 |  |
| c.3316G>A/c.3766_3767dup | 1 | 15 | 18 |  |
| c.3556+1G>A/c.3794_3797dup | 1 | 16 | 18 |  |
| c.644del/c.3796G>C | 1 | 2 | 18 |  |
| c.2620G>C/c.3799G>A | 1 | 11 | 18 |  |
| c.1820dup/c.3809A>G | 1 | 5 | 18 |  |
| c.1847G>A/c.3809A>G | 1 | 5 | 18 |  |
| c.2075T>C/c.3809A>G | 1 | 7 | 18 |  |
| c.2120A>G/c.3809A>G | 1 | 7 | 18 |  |
| c.2227del/c.3809A>G | 1 | 8 | 18 |  |
| c.2278C>T/c.3809A>G | 1 | 8 | 18 |  |
| c.2804C>T/c.3809A>G | 1 | 12 | 18 |  |
| c.2939G>A/c.3809A>G | 1 | 13 | 18 |  |
| c.3459G>T/c.3809A>G | 1 | 16 | 18 |  |
| c.3556G>A/c.3809A>G | 1 | 16 | 18 |  |
| c.2804C>T/c.3818C>A | 1 | 12 | 18 |  |
| c.3089G>A/c.3818C>A | 1 | 14 | 18 |  |
| c.3007G>A/c.3836A>G | 1 | 13 | 18 |  |
| c.3646G>A/c.3836A>G | 1 | 17 | 18 |  |
| c.122A>G/c.3842G>A | 1 | 2 | 18 |  |
| c.1449_1456del/c.3843dup | 1 | 3 | 18 |  |
| c.3056A>T/c.3843dup | 1 | 13 | 18 |  |
| c.3836A>G/c.3843dup | 1 | 18 | 18 |  |
| c.3316G>A/c.3848C>T/c.2785A>G | 1 | 15 | 18 |  |
| c.1543+1G>T/c.3884C>T | 1 | 3 | 18 |  |
| c.2230T>A/c.3884C>T | 1 | 8 | 18 |  |
| c.2333G>A/c.3884C>T | 1 | 8 | 18 |  |
| c.3008C>T/c.3884C>T | 1 | 13 | 18 |  |
| c.3079G>C/c.3884C>T | 1 | 14 | 18 |  |
| c.3443T>C/c.3884C>T | 1 | 16 | 18 |  |
| c.3532A>G/c.3884C>T | 1 | 16 | 18 |  |
| c.3646G>A/c.3884C>T | 1 | 17 | 18 |  |
| c.3700-1G>A/c.3884C>T | 1 | 18 | 18 |  |
| c.3832G>A/c.3884C>T | 1 | 18 | 18 |  |
| c.51+1G>A/c.3884C>T | 1 | 1 | 18 |  |
| c.2332C>T/c.3901dup | 1 | 8 | 18 |  |
| c.3587A>G/c.3903+2T>G | 1 | 17 | 18 |  |
| c.3326T>C/c.3903+5G>A | 1 | 15 | 18 |  |
| c.2662A>C/c.3904-3C>G | 1 | 11 | 19 |  |
| c.3932T>A/c.3932T>A | 1 | 19 | 19 |  |
| c.571_575del/c.3932T>A | 1 | 2 | 19 |  |
| c.1708-5T>G/c.3955C>T | 1 | 5 | 19 |  |
| c.3443T>C/c.3955C>T | 1 | 16 | 19 |  |
| c.3517G>A/c.3955C>T | 1 | 16 | 19 |  |
| c.3903+1G>T/c.3960G>C | 1 | 18 | 19 |  |
| c.3884C>T/c.3982G>A | 1 | 18 | 19 |  |
| c.3983C>T/c.3983C>T | 1 | 19 | 19 |  |
| c.2668G>A/c.4003G>C | 1 | 11 | 19 |  |
| c.3459G>T/c.4003G>C | 1 | 16 | 19 |  |
| c.3809A>G/c.4003G>C | 1 | 18 | 19 |  |
| c.3842G>A/c.4003G>C | 1 | 18 | 19 |  |
| c.2294A>G/c.4005_4006insTTATAATGGGTTGGG | 1 | 8 | 19 |  |
| c.4005_4006insTTATAATGGGTTGGG/c.4005_4006insTTATAATGGGTTGGG | 1 | 19 | 19 |  |
| c.3029A>C/c.4006del | 1 | 13 | 19 |  |
| c.51+2T>C/c.4006del | 1 | 1 | 19 |  |
| c.1708-1G>C/c.4014T>A | 1 | 5 | 19 |  |
| c.3446G>A/c.4014T>A | 1 | 16 | 19 |  |
| c.4044_4054delinsGGATGGGCCCATCT/c.4044_4054delinsGGATGGGCCCATCT | 1 | 20 | 20 |  |
| c.2827G>A/c.4069G>A | 1 | 12 | 20 |  |
| c.1206_1215dup/c.4072G>A | 1 | 2 | 20 |  |
| c.2620G>C/c.4075A>G | 1 | 11 | 20 |  |
| c.2659del/c.4075A>G | 1 | 11 | 20 |  |
| c.2930C>T/c.4109C>T | 1 | 13 | 20 |  |
| c.4112T>C/c.4112T>C | 1 | 20 | 20 |  |
| c.1449_1456del/c.4114C>T | 1 | 3 | 20 |  |
| c.1708-1G>C/c.4114C>T/c.1168A>G | 1 | 5 | 20 | 2 |
| c.2128G>A/c.4114C>T | 1 | 8 | 20 |  |
| c.2297C>T/c.4114C>T | 1 | 8 | 20 |  |
| c.2620G>C/c.4114C>T | 1 | 11 | 20 |  |
| c.2790_2792del/c.4114C>T | 1 | 12 | 20 |  |
| c.3052G>A/c.4114C>T/c.2785A>G | 1 | 13 | 20 |  |
| c.3443T>C/c.4114C>T | 1 | 16 | 20 |  |
| c.3450C>A/c.4114C>T | 1 | 16 | 20 |  |
| c.3532A>G/c.4114C>T | 1 | 16 | 20 |  |
| c.3971A>G/c.4114C>T | 1 | 19 | 20 |  |
| c.3982G>A/c.4114C>T | 1 | 19 | 20 |  |
| c.994G>T/c.4114C>T | 1 | 2 | 20 |  |
| c.3517G>A/c.4176dup | 1 | 16 | 21 |  |
